# Antibiotic Cycling and Antibiotic Mixing: Which One Best Mitigates Antibiotic Resistance?

**DOI:** 10.1093/molbev/msw292

**Published:** 2017-01-17

**Authors:** Robert Eric Beardmore, Rafael Peña-Miller, Fabio Gori, Jonathan Iredell

**Affiliations:** 1 Biosciences University of Exeter, Devon, United Kingdom; 2 Center for Genomic Sciences, Universidad Nacional Autonóma de México, Cuernavaca, Mexico; 3 Westmead Clinical School, Westmead Hospital, The University of Sydney, Australia

## Abstract

Can we exploit our burgeoning understanding of molecular evolution to slow the progress of drug resistance? One role of an infection clinician is exactly that: to foresee trajectories to resistance during antibiotic treatment and to hinder that evolutionary course. But can this be done at a hospital-wide scale? Clinicians and theoreticians tried to when they proposed two conflicting behavioral strategies that are expected to curb resistance evolution in the clinic, these are known as “antibiotic cycling” and “antibiotic mixing.” However, the accumulated data from clinical trials, now approaching 4 million patient days of treatment, is too variable for cycling or mixing to be deemed successful. The former implements the restriction and prioritization of different antibiotics at different times in hospitals in a manner said to “cycle” between them. In antibiotic mixing, appropriate antibiotics are allocated to patients but randomly. Mixing results in no correlation, in time or across patients, in the drugs used for treatment which is why theorists saw this as an optimal behavioral strategy. So while cycling and mixing were proposed as ways of controlling evolution, we show there is good reason why clinical datasets cannot choose between them: by re-examining the theoretical literature we show prior support for the theoretical optimality of mixing was misplaced. Our analysis is consistent with a pattern emerging in data: neither cycling or mixing is a priori better than the other at mitigating selection for antibiotic resistance in the clinic.

***Key words***: antibiotic cycling, antibiotic mixing, optimal control, stochastic models.

## Introduction

How best to use antibiotics is a question in applied evolutionary biology of the most profound importance for human health and yet it has been called a “*conceptually uninteresting”* scientific problem for evolutionary biologists (Read and Huijben 2009). Concepts of how to best treat with antibiotics remain as controversial as they are important. For instance, the public is told by medical professionals to adhere to the fully prescribed course of antibiotics to prevent resistance (Tabor 2008) but this practise is also said to “*make no sense”* (Taubes 2008; Rice 2008) based, as it is said to be, on an absence of data.

However, it is increasingly clear from molecular studies of patient infections that clinical resistance evolution occurs de novo (Mwangi et al. 2007; Blair et al. 2015). There is, therefore, a pressing need for molecular, evolutionary, and theoretical biologists to appropriate questions posed by medics and to bring them from the clinic into the laboratory, both wet and dry, where the full gamut of investigative tools can be brought to bear to provide the missing datasets that will resolve debates such as this. We take this approach with a question from evolutionary medicine that has been posed many times before where the answer is thought to be well understood. We will show it is not. The question is this: in an attempt to preserve their efficacy, should hospitals, and intensive care specialists, mix or cycle their antibiotics? It is now over 30 years ago that clinicians asked whether a strategy of antibiotic cycling might alleviate the resistance problem (Gerding and Larson 1985; McGowan 1986) and yet this remains an open problem.

Antibiotic cycling is the crop rotation idea applied to antibiotics (Kollef et al. 1997). Different antibiotics are prioritized against specific infections for a period of time, only for that period of drug prioritization to be replaced by one of restriction at a pre-determined later time, which could be many months (Brown and Nathwani 2005). It was hoped that cycling would select against resistance alleles because one particular drug would not be encountered by a pathogen during a restriction cycle and so resistance would “reverse” because of the fitness costs of being drug-resistant (Niederman 1997; Kollef et al. 1997). Michael Niederman summarized this idea in a question (Niederman 1997): *is the crop rotation of antibiotics the solution to a resistance problem in intensive care units*? Later, predictions were made using computer simulations of mathematical models of different epidemiological scenarios which claimed that cycling might reduce the incidence of drug-resistant infection no better than if we randomly allocated antibiotics to patients (Bonhoeffer et al. 1997). The latter idea has come to be known as “mixing” because drugs are mixed within the patient cohort; drugs are not necessarily “mixed” within patients. Treatments which do that are called “combination therapies” and these are used routinely in the clinic.

Comparing cycling against mixing provided a useful observation about how to measure the success of novel resistance mitigation strategies as it provided an appropriate baseline measure. But it was stated, later still, that cycling different drugs must be suboptimal because a strategy of maximal *“heterogeneous antibiotic use slows the spread of resistance”* (Bergstrom et al. 2004). This idea taken literally, of maximizing the heterogeneity of drug prescription, is now thought of as representing the de facto theoretical optimum (Levin and Bonten 2004). And clinicians state as much (Sandiumenge et al. 2006) in their work:

“*Mathematical models have shown that heterogeneous antibiotic use, defined as a balanced use of the different antimicrobials available, is the most likely way of reducing the selection pressure that leads to antibiotic resistance.*”

and (Takesue et al. 2010).

“*Heterogeneous antibiotic use has been suggested [by mathematical models] to limit the emergence of resistance*.”

However, we contend that statements like these are based on an over-generalization of what the details of the theory actually predict.

## Results

### Information is Key in a Toy Model Scenario

It is straightforward to see that antibiotic mixing cannot be the theoretical optimum, at least not in all theories. To understand why, imagine a highly simplified, toy scenario whereby medics at a clinic treat patients for infection by a particular pathogen. To simplify matters completely so this is a tractable model, we follow previous mathematical modeling studies in assuming just two drugs are available (Bergstrom et al. 2004; Bonhoeffer et al. 1997), we also assume all patients are infected and are treated. Previous theories (Bergstrom et al. 2004; Bonhoeffer et al. 1997; Levin and Bonten 2004) do not name the pathogen and nor do we. However, suppose we are told that the pathogen exhibits reduced susceptibility to “drug A” in 90% of prior patient cases but we do not have access to the diagnoses capable of telling us which individual patients these might be; again, nor do prior theories. Suppose that the same pathogen exhibits reduced susceptibility to the second drug “B” to which the pathogen exhibits resistance in only 10% of prior cases. To simplify the situation even further, we assume no AB cross resistance and no AB-combination treatments are given.

Since we know nothing of individual patient cases we are compelled to argue in terms of “patient fractions” that receive one or other drug. Now, if we give half the patients drug A and the half other receive drug B, which is a *mixing* strategy, the expected fraction of patients that receive an appropriate treatment is this: (the fraction treated with A) × (the fraction infected by a pathogen susceptible to A) + (the fraction treated with B) × (the fraction infected by a pathogen susceptible to B) =  $\frac{1}{2}\times (100-90)\%+\frac{1}{2}\times (100-10)\%=50\%$. Suppose, on the other hand, we give everyone drug B, which is not a mixing strategy, then the expected fraction of patients that receive an appropriate treatment is 0×(100−90)%+1×(100−10)%=90%, a greater value than the previous 50%. Thus the population is treated appropriately more often, given the information we have, if the drugs are not mixed within the patient cohort. Furthermore, the worst thing we can do is to give everyone drug A because then only 1×(100−90)%+0×(100−10)%=10% of patients are given the most appropriate drug.

And what of the resulting evolutionary dynamics? This simple scenario says nothing about how our strategy should change as resistance evolves. So, to remedy this we make the situation a little more general: suppose a fraction, *a*, of patients are given drug A, and therefore the fraction 1−a receive drug B. Assume an expected fraction, *p*, of patients have a drug-A resistant infection and suppose *q* have a drug-B resistant infection, where p≠q. We now seek a drug control strategy, *a*, that maximizes the likelihood of appropriateness of treatment.

**Fig. 1:**
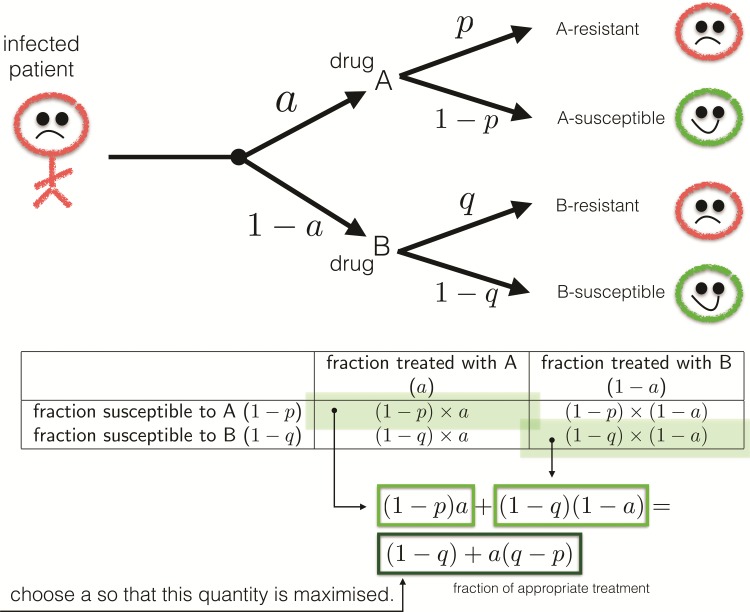
An illustration of the toy scenario. A patient seeks treatment and one of two antibiotics can be administered. If the probability of resistant infection to either drug, *p* and *q* respectively, can be estimated, optimal behavior maximizes the likelihood of appropriate therapy. This entails finding *a* that maximizes 1−q+a(p−q) where 0≤a≤1 but since this expression is linear in *a*, the maximum occurs when *a* is zero or one. However, the worst possible drug deployment protocol comes from minimizing this expression and this also arises when *a* is zero or one.

The patient fraction treated with A that exhibits a treatable, A-susceptible infection is a·(1−p), the fraction treated with A but with B-susceptibility is a·(1−q), the fraction treated with B but with A-susceptibility is (1−a)·(1−p) and the fraction treated with B exhibiting B-susceptibility is (1−a)·(1−q). The patient fraction treated with a drug that best contributes to clearance of their infection is, therefore, a(1−p)+(1−a)(1−q)=1−q+a(q−p). This calculation is illustrated in figure 1.

Antibiotic appropriateness is maximized when 1−q+a(q−p) is maximized over all possible patient fractions, *a*, which is a number between 0 and 1. Since the quantity 1−q+a(q−p) depends linearly on *a*, this means

(1) $$a=\{\begin{array}{ccc} 1 & \;\text{if}\; & q>p,\\ 0 & \;\text{if}\; & p>q. \end{array}$$

For the strategy defined in (1), max⁡(1−p,1−q) is the probability of appropriate treatment and this value is as high as we can make it given the information we have. We therefore call (1) an optimal strategy.

As we this scenario knows nothing about individual patients, according to (1) it is optimal to treat everyone with the drug for which resistance is least likely, even though this is a population-wide strategy that will be sub-optimal for some individuals. We can, therefore, improve upon this solution by getting every individual treatment decision right which means having better information on individual circumstances, from antibiograms for example, but this realistic possibility is not part of our toy scenario.

We now seek the optimal strategy in the toy scenario if the pathogens are allowed to evolve in response to our behavior as clinicians. Variables *p* and *q* will change over time as a result and we write *p*(*t*) and *q*(*t*) to allow this, *t* being time. Analogous reasoning shows that the definition of *a* in (1) should now be replaced by a time-dependent optimal strategy, $a=a_{opt}(t)$, where

(2) $$a_{opt}(t)=\{\begin{array}{ccc} 1 & \;\text{if}\; & q(t)>p(t),\\ 0 & \;\text{if}\; & p(t)>q(t),\\ \text{give}\;\text{either}\;\text{drug} & \;\text{if}\; & p(t)=q(t). \end{array}$$

Observe how $a_{opt}(t)$ cycles “reactively” between drugs depending on which of *p*(*t*) or *q*(*t*) is the greater. Note also how this strategy tacitly requires that *p*(*t*) and *q*(*t*) can be estimated at all times and that it leads to the most appropriate treatment with probability $\frac{1}{T}\int_{0}^{T}\max(1-p(t),1-q(t))dt$ if the clinic is observed for a duration of *T* time units.

The strategy $a_{opt}(t)$ has appeared before in other theories: the importance of $a_{opt}(t)$ for differential equation models of the antibiotic deployment problem was demonstrated using mathematical techniques from functional analysis (Beardmore and Pena-Miller 2010b). This idea is also contained within a family of strategies that was later termed “informed switching” for noisy differential equations (Kouyos et al. 2011). Clinically, $a_{opt}(t)$ could be likened to surveillance-based cycling where antibiotics are restricted according to patterns in emerging drug resistance data, an idea that has been trialed in the clinic (Allegranzi et al. 2002; Gerding and Larson 1985).

And what of mixing in this toy analysis? A mixing protocol, $a_{mix}(t)$, is a stochastically fluctuating, knowledge-free strategy that can fluctuate in time but whose “expected value” (that we denote E) at all times is a number between zero and one representing a constant, unchanging bias towards one of the drugs: this means $E(a_{mix}(t))=\beta$ where *β* is that biasing constant. If we are free to optimize the fraction of appropriate treatments with respect to *β* we can determine an “optimal mixing strategy” that treats appropriately with probability $\frac{1}{T}\int_{0}^{T}1-q(t)+\beta \cdot (q(t)-p(t))dt$. But, for any fixed *β*, this number is necessarily less than the optimal value $\frac{1}{T}\int_{0}^{T}\max(1-p(t),1-q(t))dt$ we gave above for $a_{opt}(t)$. So, our analysis shows, at least given the assumptions in our toy scenario, it would be wrong to mix drugs. For example, random mixing (*a.k.a.* maximal drug heterogeneity) is the strategy that sets β=1/2 which gives an appropriate treatment in a mean fraction $\frac{1}{T}\int_{0}^{T}\frac{1}{2}(1-q(t))+\frac{1}{2}(1-p(t))dt$ of cases, but this is also necessarily sub-optimal relative to $a_{opt}(t)$.

This seems clearcut, but things get interesting when we ask this: what is the worst strategy possible? Can we determine that too? This is the unfortunate case that gets the drug usage decisions wrong with maximal probability. So A (or B)-resistant infections are treated with drug A (or B) as often as possible. This occurs when we exchange *a*(*t*) with 1−a(t) in the optimal strategy above, so the worst thing possible that can be done in our toy scenario is to use the cycling strategy $a_{bad}(t):=1-a_{opt}(t)$ which gives appropriate treatment to the fraction min⁡(1−p(t),1−q(t)) of patients at any given time. It is unfortunate from a clinical perspective that the best and worst antibiotic management protocols are cycling strategies, with mixing sitting somewhere in between.

So, to summarize, our scenario manifests the following “ordering property” in terms of which behavioral strategies select for resistance by giving appropriate or inappropriate antibiotic treatments: the best strategy available ($a_{opt}(t)$) cycles drugs reactively through time and this is preferable to the best mixing strategy. But this, by definition, outperforms the worst mixing strategy which outperforms the worst strategy of all those we analyzed, namely ($a_{bad}(t)$) which, we repeat, is a reactive cycling strategy. Thus, the best possible way of cycling performs better than the mixing strategies which performs better, in turn, than the worst cycling strategy. This dichotomy with cycling and mixing is very hard to escape (Beardmore and Pena-Miller 2010b) whichever modeling paradigm we analyze, as the next section illustrates.

We first make a brief point concerning the term “optimal.” The strategy identified as optimal in the above scenario may well not achieve what we hope for. Dual resistance may well sweep through the pathogen population, it may pass to fixation and so render both our drugs impotent. It could well be that the best we can achieve is simply to slow this process because the long-term outcome we would hope to engineer (namely, to prevent the occurrence of resistance mutations) is not possible once we begin to use the drugs. In other words, “theoretically optimal” does not necessarily mean “desirable” or clinically useful.

Moreover, strategies that are optimal for one theoretical model need not be optimal for other theoretical models, let alone have value in the clinic. Indeed, it is only to be expected that different optimality criteria, and different modeling choices, will lead to different optimal strategies even in the same mathematical model. (As a technical aside, this issue is central to condensed matter physics where free energy is optimized and different optima correspond to different states of matter.) Above we used the criterion of maximizing the likelihood of appropriate treatment within a patient cohort given that everyone is treated, but different performance criteria might have asked us to compromise on this. For example, if our criterion had sought to maintain longevity of the drugs, the optimal solution could well have drawn us into a tradeoff of treating fewer infected patients (Foster and Grundmann 2006).

### A Second Toy Model

The above scenario could be criticized in many different ways for a lack of realism, but do its predictions generalize to more sophisticated mathematical models? We address this question by applying the theory of optimal control and computational tools designed to solve dynamic programming problems (Mitchell 2008) to the following differential equation model of antibiotic stewardship (Reluga 2005):

(3a) $$\frac{d}{dt}I_{1}=m_{1}+I_{1}(1-I_{1}-I_{2})-\gamma_{1}aI_{1},$$

(3b) $$\frac{d}{dt}I_{2}=m_{2}+I_{2}(1-I_{1}-I_{2})-\gamma_{2}(1-a)I_{2}.$$

Here a=a(t) is the patient fraction treated with drug 1, 1−a(t) is the fraction of patients treated with drug 2, *I*_1_ and *I*_2_ represent the densities of patients infected with drug 2- and drug 1-resistant strains, respectively, *γ*_1_ and *γ*_2_ are the clearance rates of infection when patients receive an appropriate antibiotic and *m*_1_ and *m*_2_ are the admittance rates of patients to the clinic infected by drug 2- and drug 1-resistant strains, respectively. Finally $I_{1}(0)$ and $I_{2}(0)$ are assumed known when *t* = 0.

Only the case $m_{1}=m_{2}$ and $\gamma_{1}=\gamma_{2}$ is considered in (Reluga 2005) whereas we break this symmetry, analogous to requiring that p≠q in the toy scenario, and consider other cases on the grounds of realism. It is unlikely, for example, that the rates of admittance of both patient classes *I*_1_ and *I*_2_ will be identical at all times. Moreover, if a definitive ranking of mixing and cycling were possible and clinically relevant, it would have to be robust to all reasonable variations of parameters in the model. We are therefore compelled to test whether, or not, our conclusions are robust to parameter changes in models like (3).

Now, the optimal control problem for (3) asks us to find a function *a*(*t*) so that the totality of infected patient days $\int_{0}^{T}I_{1}+I_{2}\;dt$ is minimized, where *T* > 0 is some fixed observation time. Control theory tells us that the optimal strategy can be determined by solving the so-called Hamilton-Jacobi-Bellman (HJB) equation associated with (3) numerically. This numerical approach determines optimal controls as so-called “feedback laws” whereby $a=\varphi (t,I_{1},I_{2})$ for some function φ [see (Mitchell 2008) and supplementary text, Supplementary Material online for details].

See supplementary figure S1, Supplementary Material online illustrates that when $m_{1}=m_{2}$ and $\gamma_{1}=\gamma_{2}$, the optimal control law approximates the function we determined for the toy scenario:

(4) $$a=\{\begin{array}{ccc} 1 & \;\text{if}\; & I_{1}>I_{2},\\ 0 & \;\text{if}\; & I_{1}<I_{2}. \end{array}$$

However, when $m_{1}\neq m_{2}$ or $\gamma_{1}\neq \gamma_{2}$, see supplementary figure S1, Supplementary Material online shows the optimal law can resemble the asymmetric control law

(5) $$a=\{\begin{array}{ccc} 1 & \;\text{if}\; & I_{1}>\theta \cdot I_{2},\\ 0 & \;\text{if}\; & I_{1}<\theta \cdot I_{2}. \end{array}$$

where *θ* depends on system parameters.

It must be noted that the strategies (1) and (4) do not preclude the optimality of antibiotic mixing, although this point is mathematically technical. This is because there could be theoretical cases where $I_{1}(t)\approx I_{2}(t)$ most of the time along optimal solution trajectories, in which case a near-optimal reactive control *a* would oscillate very rapidly between the deployment of either drug. While this phenomenon known as “chattering” would be irrelevant to the clinic, it could arise in theory if a mixing solution, whereby *a* = 1/2, were either very close to being optimal or else were optimal. However, as we discuss in the supplementary, this requires mathematical models to possess special symmetry properties.

### A Third Model of Antibiotic Use

Although very simple, equation (3) broaches the limits of what can be gleaned using mathematical, analytic tools. So, in order to progress, we now present two other models based on assumptions that have been articulated elsewhere (Bergstrom et al. 2004; Bonhoeffer et al. 1997) for which we cannot determine optimal antibiotic controls but that we can use to compare mixing and cycling. We will present synthetic data from both models in order to illustrate the generality of our arguments.

The first of these models, equation (6), assumes two antibiotics are available to treat infected patients, labeled 1 and 2. It assumes *S* is the proportion of patients in the hospital infected by a drug-susceptible pathogen, that *R*_1_ then represents the proportion of patients infected by a drug-1-resistant pathogen, similarly for *R*_2_, and then *X* is the proportion of uncolonized patients. The model is this:

(6a) $$\begin{array}{c} \frac{d}{dt}S=\mu (m-S)-(\tau_{1}+\tau_{2}+\gamma )S+\beta SX+\ldots \\ \ldots +\sigma \beta (c_{1}R_{1}+c_{2}R_{2})S, \end{array}$$

(6b) $$\begin{array}{c} \frac{d}{dt}R_{1}=\mu (m_{1}-R_{1})-(\tau_{2}+\gamma )R_{1}+\beta (1-c_{1})R_{1}X-\ldots \\ \;\ldots -\sigma \beta (c_{1}S+(c_{1}-c_{2})R_{2})R_{1}, \end{array}$$

(6c) $$\begin{array}{c} \frac{d}{dt}R_{2}=\mu (m_{2}-R_{2})-(\tau_{1}+\gamma )R_{2}+\beta (1-c_{2})R_{2}X-\ldots \\ \;\ldots -\sigma \beta (c_{2}S+(c_{2}-c_{1})R_{1})R_{2}, \end{array}$$

(6d) $$\begin{array}{c} \frac{d}{dt}X=\mu (1-m-m_{1}-m_{2}-X)+(\tau_{1}+\tau_{2}+\gamma )S+\ldots \\ \ldots +(\tau_{2}+\gamma )R_{1}+(\tau_{1}+\gamma )R_{2}-\ldots \\ \ldots -\beta X(S+(1-c_{1})R_{1}+(1-c_{2})R_{2}). \end{array}$$

Equation (6) contains parameters ($\mu ,\;\sigma ,\;m,\;m_{1},\;m_{2},\;\gamma ,\;\beta ,\;\alpha ,\;\tau_{\text{max}},\;c_{1}$ and *c*_2_) the meanings of which are stated in table 1. Note that (6) makes no explicit reference to mechanisms of drug resistance evolution, whether de novo mutation in the chromosome of the pathogen or else through horizontal gene transfer. Both do occur in the clinic and, we believe, are likely to require different mitigation strategies.

**Table 1:** The Meaning of the Parameters in Equation (6).

Description	Parameters
Patients enter hospital in states $S,R_{1}$ and *R*_2_ at rates $\mu m,\mu m_{1}$ and $\mu m_{2}$ respectively	$m,m_{1},m_{2}$
Rate of use of drugs 1 and 2 per unit time (days)	$\tau_{1},\tau_{2}$
Fitness cost of resistance to pathogens	*c*_1_, *c*_2_
Relative rate of secondary colonization to primary colonization	*σ*
Rate constant for colonization of uncolonized individuals	*β*
Rate of patient turnover in the hospital	*μ*
Represents physician compliance with cycling program	*α*
Untreated patients colonized by susceptible bacteria remain colonized 1/γ days on average	*γ*

Now, in (6), *τ*_1_ and *τ*_2_ represent rates of use of drugs 1 and 2, where all patients are assumed treated with one of the drugs. This results in a constraint, $\tau_{1}+\tau_{2}=\tau$, where *τ* a fixed constant that plays the role of “a” in our previous discussion. Following (Bergstrom et al. 2004) we seek a function, $\tau_{1}(t)$, which minimizes the total fraction of patient days observed with a drug-resistant infection, $\int_{0}^{T}R_{1}+R_{2}dt$, where *T* denotes a fixed observation time. This, again, is a question in optimal control but not one that can be easily solved. So, in the absence of any better strategies, we will apply the reactive cycling solution (4) to (6) and ask how it performs in relation to mixing.

Before doing this, we first generalize (6) so that the antibiotic usage protocol, $\tau_{1}(t)$, can be explicitly stochastic. This is done by introducing a random process into (6) so that $\tau_{1}(t)$ represents a noisy, time-varying deployment protocol such that $E(\tau_{1}(t))=\alpha (t)$ for each t≥0, where E(·) denotes expectation (see supplementary text, Supplementary Material online) and α(t) is some defined protocol that we expect clinicians to adhere to; α(t) could, for example, represent a mixing or a cycling protocol.

The second model we refer to is (7) below from (Bonhoeffer et al. 1997, Case III). It uses a slightly different terminological convention with “A” and “B” for the drug labels and $x=(x,y_{w},y_{a},y_{b})$. In this model, x is the density of patients uninfected by a pathogen, $y_{w}$ represents the number of patients infected by a wild-type pathogen strain, $y_{a}$ denotes the number of patients with a drug-A resistant strain, similarly for drug-B resistant $y_{b}$:

(7a) $$\begin{array}{c} \frac{dx}{dt}=\lambda -dx-b(y_{w}+y_{a}+y_{b})x+r_{w}y_{w}+r_{a}y_{a}\\ \ldots +r_{b}y_{b}+h(1-s)((f_{a}+f_{b})y_{w}+f_{a}y_{b}+f_{b}y_{a}), \end{array}$$

(7b) $$\;\;\;\;\frac{dy_{w}}{dt}=(bx-c-r_{w}-h(f_{a}+f_{b}))y_{w},$$

(7c) $$\;\;\;\;\frac{dy_{a}}{dt}=(bx-c-r_{a}-hf_{b})y_{a}+hsf_{a}y_{w},$$

(7d) $$\;\;\;\;\frac{dy_{b}}{dt}=(bx-c-r_{b}-hf_{a})y_{b}+hsf_{b}y_{w},$$

where the epidemiological parameters are $\left(\lambda ,\;d,\;c,\;h,\;r_{w},s,\;r_{a},\;r_{b},b\right)$ whose interpretation is stated in table 2. There are no multidrug-resistant strains in (7) and although that case has been considered in (Bonhoeffer et al. 1997), for brevity we do not discuss it.

**Table 2:** The Meaning of the Parameters in Equation (7).

Description	Parameters
The fraction of patients treated with antibiotic A and B	*f_a_*, *f_b_*
Recovery rates of wild-type, A-res and B-res infected hosts	$r_{w},r_{a},r_{b}$
Transmission rate of infection	*b*
Maximum rate at which patients are treated	*h*
Fraction of patients that acquire resistance when treated	*s*
Per capita death rate of uninfected hosts	*d*
Arrival rate of uninfected hosts	*λ*
Infected hosts’ death rate	*c*

Here, *f_a_* and *f_b_* represent the fraction of the population treated with drugs A and B and assuming everyone is treated, meaning $f_{a}+f_{b}=1$, following (Bonhoeffer et al. 1997) we seek a function $f_{a}(t)$ so that the total of all patient infected-days, i.e.

$$\int_{0}^{T}y_{w}+y_{a}+y_{b}\;dt,$$

is minimized.

The reactive control strategies are, in the case of (6),

$$\tau_{1}(t)=\tau \cdot a(t),\;\tau_{2}(t)=\tau \cdot (1-a(t))$$

and, in the case of (7),

$$f_{a}(t)=a(t),\;f_{b}(t)=1-a(t).$$

**Fig. 2:**
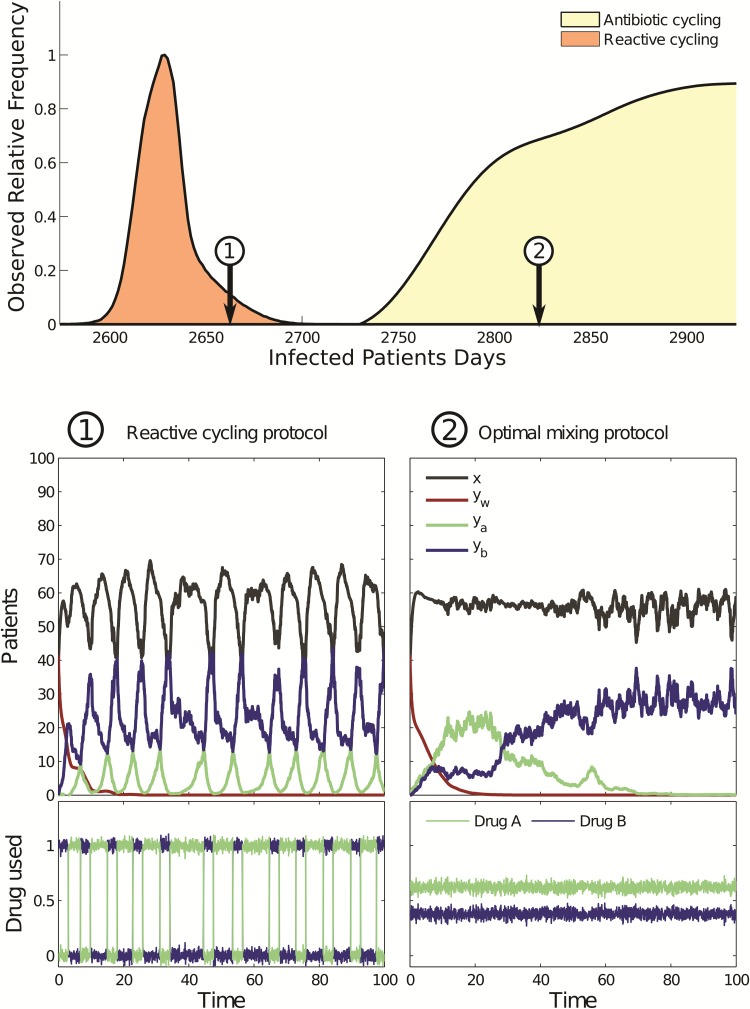
(top panel) Two performance histograms (orange and yellow, respectively) illustrate that reactive cycling can outperform all the scheduled cycling protocols. (bottom panel) Two timeseries from the stochastic version of (7) using (1, left) a protocol that cycles reactively between antibiotics and (2, right) optimal mixing: protocol (1) outperforms optimal mixing (2) as can be seen in the top panel. Variables in the legend of (2) are defined in equation (7) and performance here is the number of observed infected patients days.

Typical solutions that equation (7) produces when supplemented with this reactive control strategy are shown in figure 2 [bottom panel, see label “1.” The data for (6) are similar but are not shown for reasons of brevity]. To produce figure 2, we implemented

(8) $$a(t)=\{\begin{array}{ccc} 1 & \;\text{if}\; & y_{a}(t)<y_{b}(t)\\ 0 & \;\text{if}\; & y_{a}(t)>y_{b}(t) \end{array}$$

in (7) and this figure compares numerical solutions of the reactive cycling strategy (8) with those of the optimal mixing protocol determined for (7). They show, by example, that reactive cycling can outperform optimal mixing.

However, figure 2 gives just one comparison of optimal mixing and reactive cycling. To probe whether this comparison is representative of the general case we used a stochastic version of (7) to determine “performance histograms.” To determine performance histograms we computed the performance, namely the total number of infected patient days for a given model (i.e., with fixed parameters) for both reactive cycling and optimal mixing strategies using 10^7^ different numerical realizations of stochastic versions of (6) and (7). Figure 2 (top panel, see orange histogram and label “2”) then shows the resulting histograms. Note how reactive cycling outperforms optimal mixing in almost all the simulations of the particular model realization that we tested. (We determined performance histograms for all cycling and mixing strategies in a comparable manner, see the supplementary text, Supplementary Material online for details).

## Implications of Synthetic Data in Figure 2 for Clinical Trials

There is an important difference between the reactive cycling strategies, and cycling, used in theory and cycling in the clinic. Although some clinical studies have utilized ideas that might be described as reactive cycling (Brown and Nathwani 2005; Gerding and Larson 1985; Allegranzi et al. 2002; Takesue et al. 2010, 2006), we know of no clinical studies that implement reactive cycling in exactly the way (8) defines it. Rather, clinical trials make use of scheduled cycling protocols based on fixed periods of drug rotation (Brown and Nathwani 2005; Raymond et al. 2001; Martinez et al. 2006; Hedrick et al. 2008). So, to create a fair comparison, we must also compare (8) with the scheduled drug rotations that implement fixed, periodic cycles of antibiotic prioritization and restriction, and we must then compare this with antibiotic mixing.

Moreover, clinical cycles of antibiotic prioritization and restriction vary considerably in duration, from one month (Toltzis et al. 2002), to three (Smith et al. 2008; Hedrick et al. 2008; Warren et al. 2004) to six (Kollef et al. 1997). Indeed, many clinical cycling studies have been criticized on the basis of not implementing repeated periods of cycling (Brown and Nathwani 2005). Issues like these are difficult to avoid in practice but they are not relevant to theoretical studies where we can perform exhaustive searches using mathematical and computational models.

So, when we simulated theoretical cycling strategies based on scheduled drug rotation of fixed cycling periods, we obtained a second performance histogram, part of which can be seen in figure 2 (top panel, yellow histogram). This histogram shows the performance of entire families of cycling protocols based on a sampling of many different scheduled rotations (see supplementary text, Supplementary Material online). Importantly, figure 2 (top panel) shows that while reactive cycling outperforms scheduled rotation, many scheduled cycles outperform optimal mixing. It also appears as if the performance histogram of the family of scheduled cycling strategies contains the performance of optimal mixing right in its midst. It transpires this feature is no quirk because mathematical antibiotic deployment models, like equations (6) and (7), are compelled to have this property. For details of the argument supporting this outcome, see (Beardmore and Pena-Miller 2010a) and the supplementary.

**Fig. 3:**
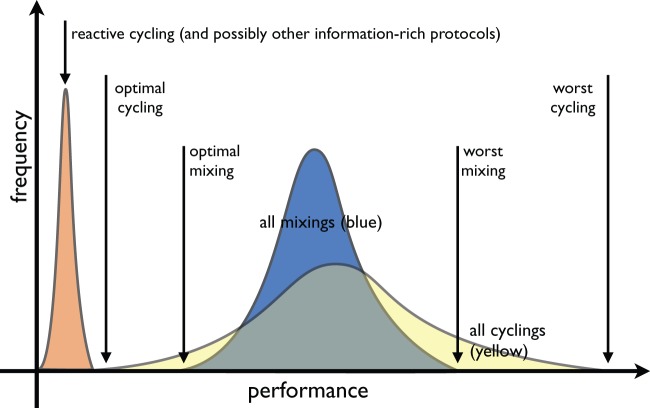
The structure of performance histograms of all cycling and all mixing protocols for a typical theoretical model. The shaded regions, each with unit area, illustrate that the range of performances of the cycling protocols is at least as wide as that of the mixing protocols and a series of numerical examples in the text illustrates this for specific models. Note that the “optimal cycling” and “optimal mixing” performance can coincide in this figure whereupon the blue and yellow histograms would have identical ranges. Note also, when illustrating this figure using mathematical model simulations in the text, we do not plot the performance histogram for all the mixing protocols, rather just optimal mixing (colored green), worst mixing (red), and random mixing (blue) are indicated.

Let us explain this feature more carefully. When differential equation models are used to examine the question of optimal antibiotic deployment, the two families of clinical strategies known as cycling (meaning scheduled rotation) and mixing (maximal antibiotic heterogeneity) must exhibit comparable levels of drug resistance in the sense that their performance distributions can always be embedded into each other, with complete overlap of their performance ranges (Beardmore and Pena-Miller 2010a). This statement is illustrated as a schematic in figure 3. It follows from this that for each mathematical model and each suboptimal antibiotic mixing protocol in it, there is a protocol which cycles antibiotics and which performs better than that mixing protocol. However, there is also another antibiotic cycling protocol with a different cadence of drug cycles which performs worse than mixing. Moreover, even if optimal mixing outperforms most cycling protocols, there are still some cycling protocols that perform nearly as well, infinitesimally close, in fact, to the performance of optimal mixing (Beardmore and Pena-Miller 2010a). So, if we were to ask which is better in general, cycling or mixing a priori, given this, it seems hard to say.

All these arguments can be summarized by a single schematic, which is figure 3. Figure 3 is derived from theoretical arguments but it is clinically relevant and it says this: if one were to randomly select just one antibiotic mixing protocol and one cycling protocol and implement both within the same mathematical model, as if one were making a clinical trial comparison where very few such comparisons are possible, one could not be a priori certain (i.e., before the models are simulated) which strategy will select most against resistance a posteriori. In a clinical context, where a limited number of trial conditions can be tested, in practise just a handful with no chance of determining performance histograms, which cycling cadence or mixing strategy *should* we choose? This decision is critical to ultimate performance of that trial but there is no way of knowing how to optimize this choice a priori, just as there is no way of doing so in a mathematical model comparing mixing and cycling.

**Fig. 4:**
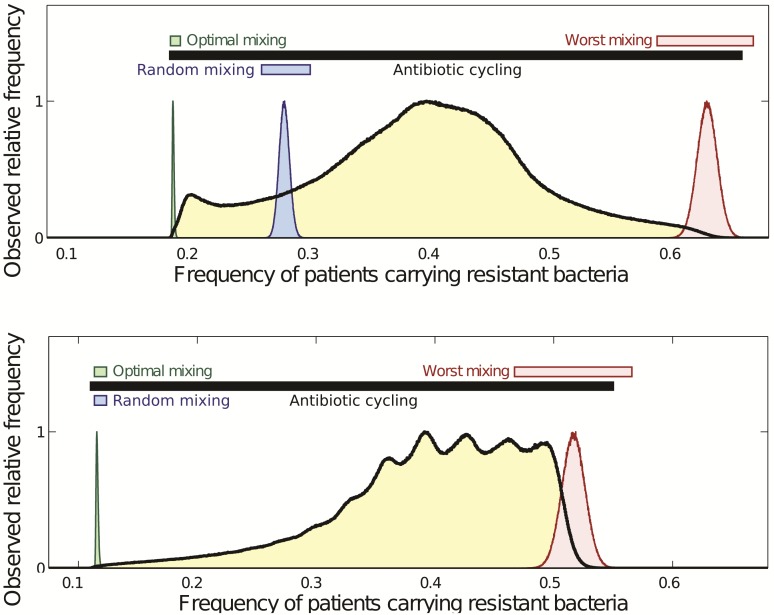
Top and bottom panels show two sets of performance histograms for (6) for cycling (10^7^ simulations in total) and three mixing protocols (10,000 simulations each). Consistent with Figure 3, both panels exhibit complete overlap in performances as indicated by the horizontal bars. While both use simulation data from equation (6), the top panel has different costs of resistance from the bottom panel that uses “symmetric” parameter values that bias in favor of random mixing. In the latter, as the bottom panel shows, there is a low probability of finding a cycling protocol that can approach the performance of random mixing, although some do. Note, this figure does not show the performance histogram for all mixing protocols in the manner done in Figure 3, rather histograms determined from stochastic simulations of (6) are shown for optimal mixing (green), the worst possible mixing (red), and random mixing (blue). Here, (bottom) the blue and green histograms coincide so only the green one is visible.

So why did prior theoretical studies (Bergstrom et al. 2004; Bonhoeffer et al. 1997; Levin and Bonten 2004) conclude that antibiotic mixing was optimal, both in theory and for the clinic? First, those studies only showed that mixing could outperform cycling in exemplar simulations, this does not show mixing is an optimal strategy, although it does show that cycling is not. Another potential answer is an unintentional bias: prior studies biased the way parameter sets were chosen with an outcome that is illustrated in figure 4 (bottom).

To explain figure 4 suppose, for sake of argument, that a mathematical model contains “rate of recovery when treated” parameters, *ρ*_1_ and *ρ*_2_ for either drug, say, but suppose that $\rho_{1}=\rho_{2}$ is assumed. Numerical assumptions like this *were* used in prior studies because an argument can be made that such assumptions engender a parsimonious, like-for-like comparison between two antibiotics with identical clinical effects whereby the only point of difference imposed in that model is that one cycles the drugs and another mixes them. This argument does seem appropriate to the question we are addressing, but it also leads to mathematical symmetries (Beardmore and Pena-Miller 2010b) that produce a systemic bias, as illustrated figure 4, that is not representative of optimal antibiotic deployment solutions in the general case. This kind of symmetry was already an issue in the toy scenario above whereby we needed a particular inequality, p≠q, in order to avoid a triviality in that discussion.

Please note that figure 4, and the remaining figures, do not show the performance histogram for all mixing protocols in the manner done in figure 3, rather they just show histograms for optimal mixing (colored green throughout), the worst possible mixing (in red) and random mixing (in blue) using 10^4^ stochastic model simulations. Performance histograms for mixing protocols are depicted alongside horizontal bars that illustrate the numerical range of 10^4^ stochastic simulations for that mixing protocol, where all simulations are performed with the same noise parameter (see supplementary text, Supplementary Material online).

While figure 3 is merely a schematic showing cycling and mixing are impossible to separate in terms of their overall performance, the structure of this figure is readily observed in specific mathematical models when we compute performance histograms, as figure 4 illustrates. Figure 4 also shows that while figure 3*is* representative of model outcomes irrespective of particular parameter choices, those choices can happen to skew in favor of random mixing, and against cycling, if parameters are chosen to have the symmetries we mention above. For instance, if we impose a symmetry of the form $m_{1}=m_{2},r_{1}=r_{2}$ and $c_{1}=c_{2}$ in (6) we can skew our computations in favor of mixing. The mathematics behind these symmetries are discussed in detail in (Beardmore and Pena-Miller 2010b).

Figure 4 (top) shows a second realization of equation (6) that uses different numerical values for the parameters defined in table 1. This figure still highlights how the overlapping nature of the performance distributions of mixing and scheduled cycling is present, just as it should be according to figure 3. However, the choice of parameter values when mixing was said to be optimal (Bergstrom et al. 2004) are symmetric and have skewed the performance distribution [fig. 4 (bottom)]. This choice ensures highly-performing cycling strategies are rare and cycles that are highly performing exchange the drugs unfeasibly quickly.

**Fig. 5:**
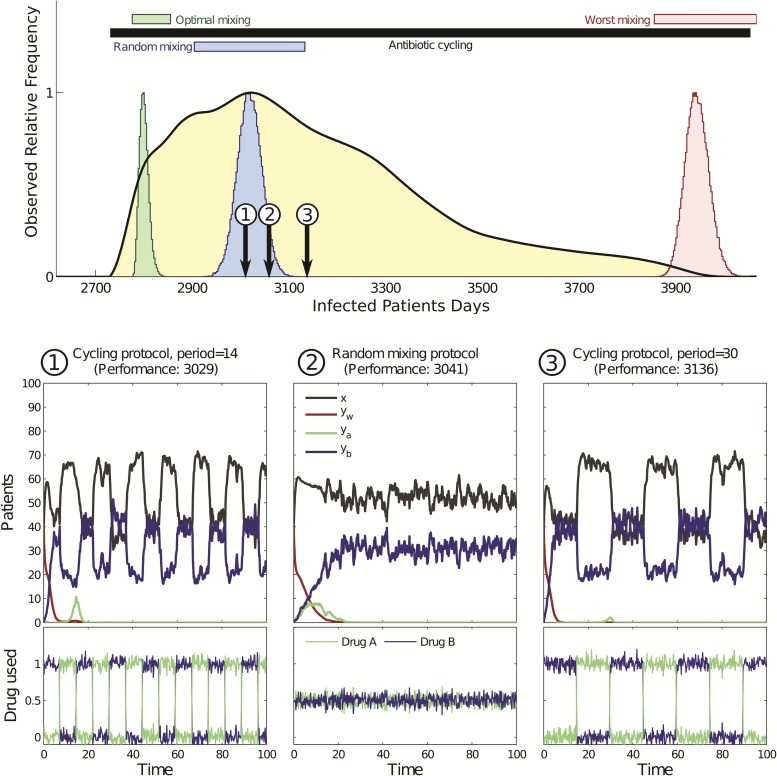
(top) This illustrates Figure 3 using performance histograms determined from equation (7), although only *three* mixing histograms are shown (random, optimal, and worst). The range of performances, from the best to worst mixings, and the range of the cyclings are shown as horizontal bars: note how they overlap consistent with Figure 3. (bottom) The random mixing protocol (indicated with a 2 in top and bottom panels) is deployed into a stochastic version of (7) and it outperforms one cycling protocol (label 3) but it under performs another cycling (label 1). This ordering property is a general feature of theoretical models like (7).

To further illustrate that our conclusions, as embodied in figure 3, are not model-specific, we present figure 5 determined using equation (7). Consistent with figure 3, the performance histograms of mixing and cycling (i.e., scheduled rotation) protocols are embedded within each other. For equation (7) the definition of performance is different, there it is the total number of infected patient days (Bonhoeffer et al. 1997), and yet the properties of the ranges of the performance distributions of mixing and cycling are just as before, they overlap.

## Discussion

Despite the problems, theory is useful when contrasting antibiotic mixing and cycling. The manifold decisions made when designing a clinical trial are almost impossible to standardize, but one can standardize mathematical models. This is important. For example, suppose the drug order of cefepime, ciprofloxacin, piperacillin-tazobactam, and imipenem-cilastatin in the quarterly cycles described to tackle drug-resistant *Pseudomonas aeruginosa* in (Hedrick et al. 2008) had been different? Suppose the trial had instead restricted ciprofloxacin entirely and implemented twice-yearly cycles? What then? The possibilities are almost limitless. How could one even hope to implement the various empirical controls needed to understand how such changes would impact on resistance evolution in the clinic? This lack of standardization is seen by clinicians as a driver behind some of the problems in answering Niederman’s question (Brown and Nathwani 2005). However, we have shown that determining a priori which of cycling and mixing selects best against drug resistant pathogens is still not possible, even when we carefully standardize the questions using mathematical models.

Intriguingly, deterministic and stochastic models of the antibiotic deployment problem have been said to have different optimal strategies: deterministic models have mixing strategies, stochastic models have switching strategies (Kouyos et al. 2011). As our derivations of treatments that outperform mixing apply equally, whether or not the models are stochastic, there is no such dichotomy here and the theoretical reasons supporting the existence of such a dichotomy are not clear. Instead, here, we find the key theoretical issue is how much clinical and microbiological information each resistance mitigation strategy is able to exploit.

**Fig. 6:**
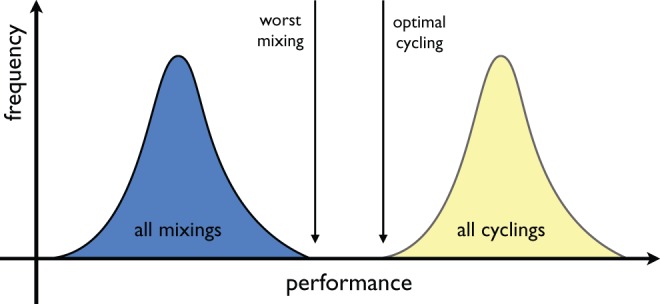
A schematic of impossible performance histograms. If this outcome were possible in a theoretical model, we would then be certain that mixing antibiotics outperforms cycling in a mathematical model, but it has been shown (Beardmore and Pena-Miller 2010b,a) that this arrangement of histograms cannot arise in equations of the form (6) and (7).

A tacit expectation in this field of study is a, quite reasonable, hope that the construction of theoretical epidemiological models will definitively resolve questions on how we should act in the clinic to prevent antibiotic resistance evolution. But why should this be so? If the expectation of modelers had been that we might identify circumstances in which, say, figure 6 were possible, whereby mixing is definitively optimal, then, unfortunately, this is not the case (Beardmore and Pena-Miller 2010b,a) at least not when using ‘SI models’ commonly applied in mathematical epidemiology. And equations (6) and (7)*are* SI models.

Ultimately, the technical, mathematical reason (Beardmore and Pena-Miller 2010b) for the impossibility of figure 6 is this: constant functions (aka mixing) controls can be approximated by oscillatory functions (aka cycling) as closely as we like in “weak topologies” of spaces of antibiotic control functions. When the equations of SI models, and their performance criteria, define continuous mappings with respect to those topologies figure 6 cannot apply. Thus, even if mixing were optimal in such a model, we could do just as well by cycling drugs quickly and this forms the main idea for the mathematical proof (Beardmore and Pena-Miller 2010a) behind figure 3. Whether this has any relevance to the clinic depends on what the term “quickly” means in practise. Indeed, one can cycle drugs every day in a model, one cannot do that in the clinic.

So, mathematic models tell us that some of the difficulties of comparing mixing and cycling are inherent to the theory behind Niederman’s question. They cannot be resolved, not even within the solution sets of certain mathematical models, be they deterministic or stochastic. We should not, therefore, be surprised when clinical trials designed to rank cycling and mixing also prove inconclusive. After all, clinical trials cannot even begin to resolve performance distributions. This uncertainty likely contributes to the ongoing community debates as to the relative merits of cycling and mixing (see http://goo.gl/Ztywjg; last accessed January 4, 2017). Meanwhile, trials continue, like the recent multinational study, the Saturn Project, that is said to be designed to “*resolve an issue of high controversy (antibiotic cycling vs. mixing)”* (see http://www.saturn-project.eu; last accessed January 4, 2017), whose authors recently stated this about their data:

‘*…there were no statistically significant differences in the prevalence of antibiotic resistance during mixing and cycling interventions*.’

We propose that figure 3 might provide a theoretical explanation of this statement.

Like cycling, mixing has been tested in at least three prior clinical studies (Takesue et al. 2006, 2010; Ginn et al. 2012). It did contribute to a reduction in resistant Gram-negative infections in a hospital-wide study (Takesue et al. 2010) but fared less well when implemented in an intensive care unit (Takesue et al. 2006). It was partially successful in one study where it may have contributed to a reduction in MRSA infection, but without impacting on Gram-negative infections (Schultsz et al. 2013). However, such positive outcomes might equally be attributed to pathogen-specific measures as to mixing, such as the reduction of *carbapenem* usage correlating with reduced carbapenem resistance in *Pseudomonas aeruginosa* (Takesue et al. 2010) or the introduction of infection control measures known to be effective in limiting fomite-spreading pathogens like MRSA (Schultsz et al. 2013). Indeed, a recent clinical study concluded (Ginn et al. 2012) that “*…prescribing homogeneity per se does not appear to be a specific resistance driver*.” One analysis of over 3.5M patient-days of antibiotic use data across 42 hospitals “*found no significant relationship between [antibiotic] diversity and the proportion of resistant pathogens”* (Pakyz et al. 2008).

It is not straightforward to find consistency in the available clinical data (McGowan 1986; Brown and Nathwani 2005; John 2000) and, in summary, there is data both for (Sandiumenge et al. 2006; Takesue et al. 2010) and against mixing (Takesue et al. 2006) but the same can be said of cycling. The support for cycling (Raymond et al. 2001; Martinez et al. 2006; John 2000) is tempered by others who advocate against it, or who at least indicate their indifference to it (van Loon et al. 2005; Warren et al. 2004; Toltzis et al. 2002). To give one more example, the cycling of linezolid and vancomycin in an ICU was called a “*promising method to reduce infections with MRSA”* (Smith et al. 2008) but cycling was also implicated as the cause of an outbreak of multi-drug resistant *Pseudomonas aeruginosa* (Hedrick et al. 2008).

Cycling clearly cannot work if resistance to antibiotics is not lost after a drug is withdrawn (Enne et al. 2001) or if it returns to baseline levels soon after drugs are reinstated (Brown and Nathwani 2005). In all this variation, a concensus has emerged describing the body of trial data as “inconclusive” (Kouyos et al. 2011). From our analysis of the theory, and given figure 3, this variation is not surprising.

### An Individual-Based Treatment Model

As a dénouement, we sought a way of bringing the individual patient into this discussion. It is individuals that we treat with antibiotics and yet, to our knowledge, no theoretical treatment of mixing deals with individuals. Patient-specific evolution occurs during treatment (Mwangi et al. 2007; Blair et al. 2015) and this may mean that individualized treatments (pathogen-specific and host-specific) will be necessary to properly optimize antibiotic use. Indeed, the FDA has approved devices that can target infections based on a rapid diagnosis of the pathogen from molecular signatures or blood cultures (Sullivan et al. 2013; Jung et al. 2014; Bergeron and Ouellette 1998a,b), including devices for *Clostridium difficile* (Ber 2008). We predict that if these approaches are considered within mathematical studies, mixing will not be the optimal way of using antibiotics there either because the principle that better decisions accrue from better information (Beardmore and Pena-Miller 2010b) will apply.

**Fig. 7:**
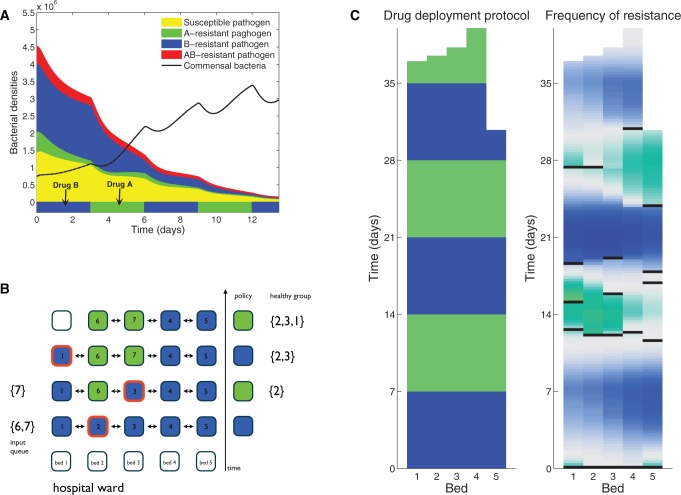
Illustrating the individual-based model. (*a*) This shows “health state dynamics” of a patient during a sequential antibiotic treatment: blue and green colored areas show the drug used and the subsequent load of drug-resistant and drug-susceptible pathogens in the host through time. The black line is the density of a commensal bacterium that competes with the pathogen for resources. The patients is deemed “recovered” when the commensals have outcompeted the pathogen. (*b*) This shows treatments in a ward of five beds where a queue of seven patients (labeled {1,2,3,4,5,6,7}) is to be treated. Colored boxes indicate the protocol where green and blue boxes, respectively, denote patients only treated with one of the two available drugs, a red outline denotes the discharge of a recovered patient and the arrival of a new patient. (*c*) An illustration of the spatial structure in the ward resulting from one realization of the model using a queue of 20 patients and five beds where a seven-day cycling protocol has been implemented, blue and green colors represent different drugs used. The relative frequency of each of the two single-drug resistant pathogens in each patient is shown in the right-most skyscraper illustrating that drug resistance is correlated with the drug usage policy. Gray regions are hosts carrying equal fractions of two different single-drug resistant pathogens, blue and green colors indicate that one of the drug resistant strains dominates the infection of the host in that bed.

To test this, we implemented an agent-based computational model (see supplementary text, Supplementary Material online) in which a much-simplified hospital ward contains an array of “beds” and a randomly ordered “queue” of patients is treated until all have recovered (fig. 7). When admitted to the ward, patients are infected with a community-acquired pathogen that shares an ecological niche and engages in competition with a commensal bacterium within the host. We do not name the pathogen, it is merely a simulation of a bacterium in a framework for comparing resistance mitigation strategies where the unit of treatment are individuals rather than population classes.

The pathogen causes illness but it is not life threatening and the commensal will eventually outcompete the pathogen (fig. 7*a*). However, the rate of patient recovery can be increased by treating appropriately with one of two antibiotics and patients are discharged when their infection is deemed to have reached an “asymptomatic” density threshold. A patient from the queue is then assigned to the newly-freed bed and treated. Different protocols are ranked using the mean length of stay (LoS) statistic for each queue where shorter LoS statistics represent better performance.

### Different Treatment Strategies in the Clinic and the Individual-Based Model

We implemented several antibiotic stewardship protocols in this model following the many strategies implemented in practise: cycling (Raymond et al. 2001; Martinez et al. 2006; Hedrick et al. 2008); mixing based on the adjustment of future prescription patterns by monitoring prior prescription data (Takesue et al. 2006, 2010; Sandiumenge et al. 2006); surveillance-based cycling (Allegranzi et al. 2002; Gerding and Larson 1985); rapid DNA-based diagnoses with an appropriate drug then given (Bergeron and Ouellette 1998a,b); patient-by-patient rotation of antibiotics (Sandiumenge et al. 2006) and, finally, the de-escalation of a combination therapy (Smith et al. 2008; Ame 2005). For a review of other clinical protocols, we refer to (MacDougall and Polk 2005).

A “reactive mixing” protocol that turns prior infection data into future stewardship practise has also been evaluated in the clinic: the *PAMS* methodology (“periodic antibacterial monitoring and supervision”) maximizes antibiotic heterogeneity because drugs used in the past gain a low probability of being used in the future (Takesue et al. 2006). A numerical index, essentially information entropy, measures antibiotic heterogeneity and prescription maximizes this index as time progresses. PAMS does not account for patterns of resistance that emerge during a trial, but it is hoped it will respond to patterns of drug use that might correlate with resistance.

Based on this survey, the following protocols were simulated in the individual-based model:

1. *random sequential treatment*: each patient receives a random drug each day (extreme mixing that maximizes drug heterogeneity);
2. *empirical treatment*: a random drug is allocated to the patient but not changed thereafter (also mixing);
3. *scheduled rotation* (*periodic cycling*): cycles of prioritization and restriction are fixed before any patients are admitted;
4. *periodic antibiotic monitoring and supervision* (PAMS): the next patient admitted is treated with the antibiotic that maximizes the heterogeneity of drugs used so far (mixing);
5. *surveillance-based rotation*: the drug estimated to have the lowest current prevalence of resistance from recent antibiograms is prescribed to all patients (the strategy from the toy scenario);
6. *Personalized(DNA-based) treatments*: a rapid assessment is made of the genotype responsible for infection for every patient. Reassessments conducted during treatment yield a sequential monotherapy that maximizes drug appropriateness at all times, even if resistance emerges in the host during treatment.

We do include multidrug resistance in this model but we do not implement combination therapy as it is not clear how to engineer a fair, like-for-like comparison of the performance of drug combinations in a theoretical test of mixing and cycling.

### Individual-Based Model Outcomes

**Fig. 8:**
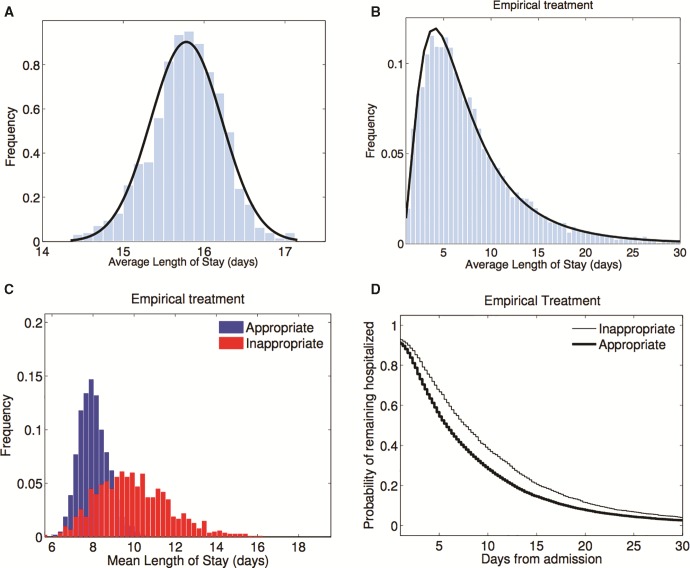
Individual model LoS statistics with no treatment and empirical treatment. (*a*) The mean length-of-stay distribution (LoS) when no patient is treated with antibiotics is a normal distribution with mean close to 16 days for the parameter values implemented (see supplementary text, Supplementary Material online). (*b*) The LoS distribution when treating empirically follows a log-normal distribution in the same conditions. (*c*) We grouped patients treated with empirical therapy into two a posteriori classes: appropriate and inappropriate, according to the drug administered. Accordingly, the LoS distribution when drug use is *inappropriate* in empirical therapies have both higher mean and variance. Inappropriate drug allocation is therefore responsible for the large variance of the empirical treatment strategy in (*b*). (*d*) For illustrative purposes: a Kaplan-Meier plot illustrating the LoS for the two classes from (*c*).

In the absence of treatment, the length of stay (LoS) data are normally distributed whereas they are log-normally distributed when everyone is treated using empirical therapy (fig. 8). Interestingly, empirical treatment reduces the mean LoS relative to treating no-one, but it also increases the LoS variance (fig. 8). So, although most patients fare better when treated, empirical treatment exhibits the following tragedy of the commons (Foster and Grundmann 2006): as resistance spreads, some patients fare worse when everyone is treated empirically than if nobody had been treated. Empirical treatment of course works well if it allocates the correct drug, which happens by chance in 50% of cases, but the LoS data increases when an inappropriate drug is administered, also in 50% of cases (fig. 8*b*–*d*). This is why treating empirically has such a large variance in its LoS data.

**Fig. 9:**
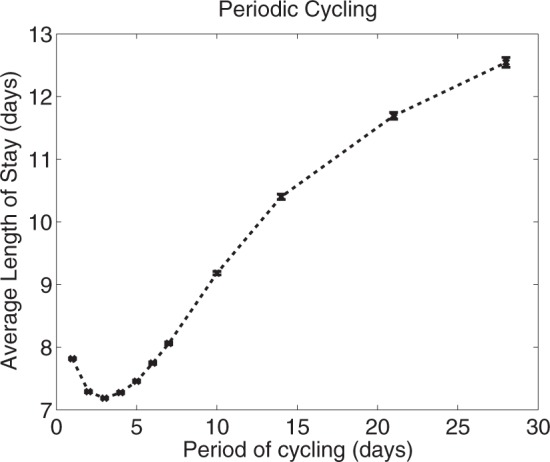
The optimal cycling period for this one particular instance of the individual patient model is very short at about 3 days. That it is so small relative to real-world clinical trials is likely due to the rapid turnover of patients in the model and the fact that there is no evolution of resistance among pathogens in the community that supplies patients to the model.

**Fig. 10:**
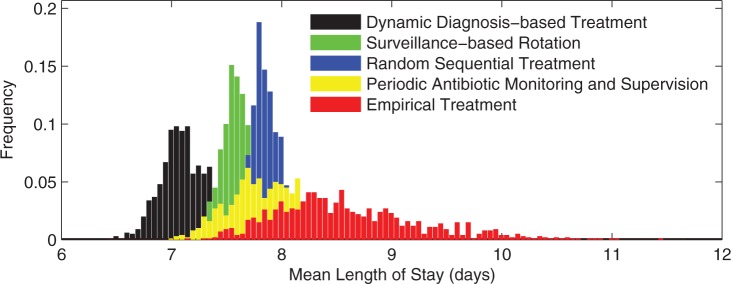
We can improve upon mixing and cycling cadences by rapidly diagnosing the pathogen genotype responsible for infection. LoS distributions for different strategies show the optimal treatment exploits most of the available information (the rapid, DNA-based, diagnosis strategy). Empirical treatment, using no information at all, is the worst performing strategy here, although it is preferable to cycling with very long cycling times (see fig. 9).

Figure 9 shows this model is consistent with the idea that there is a sweet spot LoS with respect to antibiotic cycling cadence, so that cycling works best when neither too fast nor too slow. However, determining the optimal cycling cadence so we can profit from this observation would be unfeasible in practise. Also note how the LoS at the sweet spot in figure 9 performs in relation to other strategies in figure 10: an LoS of about 7 days means optimal cycling can perform just as well as any protocol. However, other cycling strategies also perform poorly, as bad as having a LoS of two weeks when the drugs are cycled too slowly.

**Fig. 11:**
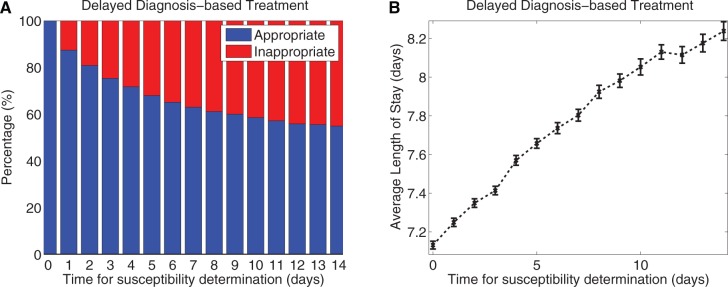
Delaying the availability of pathogen genotype information leads to deterioration in performance of the patient-specific treatment. (*a*) Delaying the availability of pathogen drug-susceptibility information decreases drug appropriateness to about 80% of patients with a 2-day delay and to no better than random (i.e. empirical treatment) with very long delays. (*b*) Consistent with this, long delays increase the expected LoS and give this protocol performances close to empirical treatment (shown are LoS means ± 95% CI from 1000 model simulations).

Figure 10 summarizes our final main result: one cannot rank strategies 1–6 outlined above definitively because their LoS distributions overlap. Other outcomes are possible in differently parameterized instances of this model, but the set of simulations shown in figure 10 indicates the patient-specific treatment (strategy 6) can sometimes produce the lowest mean LoS. Delays in using DNA tests to determine the pathogen can decrease performance. For instance, if, during the delay, empirical therapy is given, a delay of 2 days decreases drug appropriateness to 80% [see fig. 11*a*), this model outcome is consistent with values observed in the clinic (Shorr et al. 2008)] and LoS performance deteriorates towards that of empirical therapy as the delay increases further (fig. 11*b*). Finally, surveillance-based rotation (that according to the toy scenario should approximate optimal cycling) and PAMS (a reactive form of mixing) have similar performance in this model (fig. 10).

We conclude that information-rich, personalized protocols can outperform antibiotic cycling and mixing in some mathematical models but, we should emphasize, this conclusion will depend on nuanced model circumstances. For example, if all patients are infected with pathogens susceptible to both drugs, a personalized strategy will not outperform mixing because any treatment will be successful. At the other extreme in terms of the community prevalence of resistance, if multi-drug resistance has fixed in the pathogen in the community and so is present in all infections before patients begin their treatment, it will also matter little which treatment patients are given because none will work. However, before that stark situation arises, and somewhere in between these two extremes, our simulation data shows that targeting appropriate treatments at as many individuals as possible can outperform both mixing and cycling.

## Supplementary Material

Supplementary data are available at *Molecular Biology and Evolution* online.

## Supplementary Material

Supplementary Data
