# Supplementary material for: Antibiotic Cycling and Antibiotic Mixing: Which One Best Mitigates Antibiotic Resistance?
Source: Mol Biol Evol. 2017 Jan 17;34(4):802–17. doi: 10.1093/molbev/msw292 (PMC5400377; doi:10.1093/molbev/msw292)
Supplement: Supplementary Data [file msw292_Supp.pdf]

ANTIBIOTIC CYCLING AND ANTIBIOTIC MIXING: WHICH ONE BEST MITIGATES ANTIBIOTIC RESISTANCE?

R. E. BEARDMORE, R. PEÑA-MILLER, F. GORI AND J. IREDELL

1. Background and context

Mathematical models have been presented in the literature in an attempt to understand the best way of deploying antibiotics in hospitals and intensive care units [5, 3, 10]. Their main prediction for managing clinical infections is that antibiotic prescription should maximise the heterogeneity of antibiotics found in the ward, or hospital, through time and space. This strategy for managing resistance is known as 'antibiotic mixing'. This prediction has been tested and the datasets across several clinical trials are variable and inconclusive, with [13] stating that there is '*no significant relationship between [antibiotic] diversity and the proportion of resistant pathogens*' from an analysis of several million patient days of treatment.

Our article asks why what appears to be a definitive prediction from mathematical models should not be in agreement with trial outcomes, particularly as the trials continue (`http://www.saturn-project.eu`). Importantly, the authors of the latter trial state the following about their data:

> '*...there were no statistically significant differences in the prevalence of antibiotic resistance during mixing and cycling interventions.*'

(see `http://www.saturn-project.eu/main-findings-results/`) and we contend that this paper provides a potential theoretical explanation of this outcome.

Mathematical modellers have tested antibiotic mixing against a strategy known as 'antibiotic cycling', this is the crop rotation idea applied to antibiotics [9]. It is implemented by having different antibiotics prioritised against specific infections for a period of time, only for that period of prioritisation to be replaced by one of restriction at some pre-determined later time, which could be many months [6]. As the citations in the main text show, cycling, like mixing, does not necessarily perform well in clinical trials.

For this reason, we revisit the original mathematical models and ask, was the correct inference made that mixing was the optimal antibiotic stewardship strategy? After all, it is possible, in principle, to write down many different mathematical models of the antibiotic deployment problem and to analyse those models in myriad ways. Even given one model, take any from [5, 3], it will contain many biological and epidemiological parameters and we would hope that any theoretical prediction stated for the clinic were robust to uncertainties in those model choices and their parameters, particularly as many of these cannot be determined accurately, if at all, in practise. Moreover, predictions from models must be robust against both host and pathogen heterogeneities if they are to be successful in the clinic, so our article asks this: are prior findings robust? Our main result is that predictions about the optimality of mixing are not robust in a variety of ways.

All models rest on a set of core assumptions and it is inevitable, given the difficulties of model-building, that some of these do not respect many features of hospital treatments: only

two or three antibiotics are assumed available in prior theories; the models do not mimic individual patient responses, whether those described by patient genetics or general patient health (such as their age or body-mass index); within-host evolution during treatment is ignored; details of the microbiome and host immunity are absent; common clinical practices in terms of changes in drug regimen are not represented, like the de-escalated, broad spectrum empirical therapy used in the clinic [11].

The purpose of our article is to show that this broad lack of detail matters, as do more subtle changes in model assumptions. These changes in assumptions and in the values of model parameters that come with them, are enough to ensure that the applicability of prior conclusions regarding the optimality of mixing [5, 3, 10] are clinically irrelevant. This is because those conclusions are, at best, mathematically relevant only in a very narrow (ie. infinitessimal) window in model and parameter space [2].

To be clear, when we re-analyse prior models, mixing does not solve the optimal antibiotic stewardship problem. Here, we show that one can use optimal control-theoretic techniques to understand what might, instead, solve it, but it is difficult to interpret these theoretical solutions in a manner that applies to the clinic. However, it is clear that feedback controls that take information about the dynamics of the model under study and so produce adaptive control responses that give the appropriate antibiotic according to context certainly can outperform mixing. And they outperform antibiotic cycling too [2].

Moreover, cycling and mixing have a theoretical property which makes their performance hard to compare in a definitive way, even in theoretical models [1]. In the latter reference it has been proven that, for a given mathematical model and for a fixed model parameter set, however good or bad a particular sub-optimal mixing strategy is, there is always a cycling strategy that performs better and there is one that performs worse than that mixing strategy. In other words, it would be wrong to state *a priori*, before knowing model parameters, that 'mixing outperforms cycling' or 'cycling outperforms mixing'. One can only work out which one is better *after* model parameters have been fixed and then by simulating the mathematical model on a computer (or solving it by some other means). This means statements concluding that mixing is optimal must be inapplicable to the clinic, unless we are truly fortunate, because that statement is not even true for all mathematical models. There are model situations whereby optimal mixing outperforms all cycling strategies (i.e. all those based on scheduled rotation), but even in this case, cycling quickly enough in the theoretical model will always perform just as well as mixing.

Although this seems a negative outcome in terms of solving the mathematical problem at hand of finding the best way of deploying antibiotics, we define a computational model of this problem which, unlike prior models, encodes individual patients in a 'ward' and it allows pathogens to diffuse explicitly in that ward. In this model, we find that mixing can be bettered in appropriate metrics (namely, mean patient length of hospital stay) by an intuitive, almost obvious, solution. This solution is the idea that if clinicians determine, for as many patients as possible, and as soon as possible, which pathogen the patient is infected with, which drugs it is resistant to, and if they provide an appropriate drug at that point, namely

one to which the pathogen is susceptible, that is the best that one can do. This approach minimises the mean length of stay of patients in the ward in our model simulations.

Does this prevent resistance in our models? No, the use of antibiotics has the unfortunate side effect that their use leads to the emergence and spread of resistance. We cannot halt that in models unless drug resistance gene mutation rates are set to zero. But it is clear that the random use of antibiotics, aka mixing, without accounting for patient-specific molecular information about their infection, is either 1) not the optimal way of using drugs in any model in this paper or, 2) in those very rare model cases where it is optimal, other strategies perform just as well as mixing.

2. Background mathematical arguments

2.1. **Generic mathematical structure: the first modelling triviality to avoid.** Many mathematical models used to study the antibiotic stewardship problem can be written in the following form. First, a vector, $\mathbf{x}$, is used as a placeholder for all the features of the hospital ward that might be deemed relevant to the question, which features are used is a modeller's choice. One then formulates a dynamical system

$$\frac{d}{dt}\mathbf{x} = f(\mathbf{x}), \qquad \mathbf{x}(0) \text{ given},$$

which describes the ward in the absence of antibiotics. One then modifies this equation to represent changes that arise to these dynamics when one antibiotic is used. There are many ways of doing this, but the following structure is useful:

$$\frac{d}{dt}\mathbf{x} = f(\mathbf{x}) + A(t)g(\mathbf{x}).$$

This says that a function g controls features like the dynamics of treatment, patient recovery and the onset of resistance when an antibiotic drug, call it drug A, is used at some rate, $A(t)$ per unit time. However, when there are two drugs we can include a second one in an analogous manner by writing

$$\frac{d}{dt}\mathbf{x} = f(\mathbf{x}) + A(t)g(\mathbf{x}) + B(t)h(\mathbf{x}).$$

This equation tells us that B is a function controlling the rate at which drug B is used and h is another function which details the epidemiological dynamics of the ward when it is used. Given this context, $A(t)$ and $B(t)$ are unknowns to be determined that represent antibiotic stewardship protocols, namely the fraction of patients treated with each of the drugs.

If one assumes that that everyone is treated with one of the drugs, this yields a constraint on A and B of the form $A(t) + B(t) = 1$ for all t. We therefore obtain a differential equation with the following 'control-affine' structure

$$\frac{d}{dt}\mathbf{x} = f(\mathbf{x}) + h(\mathbf{x}) + A(t)(g(\mathbf{x}) - h(\mathbf{x})) \tag{1}$$

which has $A(t)$ within in it as a control variable provided g and h are different functions. The requirement that there are different functions is analogous to the requirement in the main text that $p \neq q$ in toy scenario. This assumption is used there to ensure the antibiotics modelled are distinct in a way that avoids certain mathematical trivialities. If they were not distinct it would be irrelevant how the antibiotics are managed in hospital because (1)

would say that the performance of the model is independent of how they are managed, this is clearly one triviality that needs to be avoided in this problem. This modelling assumption is akin to asking that the two antibiotics effect the dynamics of the ward differently, as would likely be the case if they were taken from two different drug classes.

For notational convenience we may as well write equation (1) in the form

$$\frac{d}{dt}\mathbf{x} = F(\mathbf{x}) + A(t)G(\mathbf{x}), \tag{2}$$

where $F = f + h$ and $G = g - h$ and this equation can be solved numerically for a given antibiotic deployment protocol, $A(t)$. If we supplement the model with a performance functional

$$\mathcal{P} := \int_0^T (\mathbf{x}(t), \mathbf{w})dt,$$

for some weight vector, $\mathbf{w}$, we can ask pertinent questions, for instance 'which antibiotic management strategy, $A(t)$, maximises performance, $\mathcal{P}$'?

3. Two Exemplar Models

The first example within this modelling framework comes from [3] in which two antibiotics are available to treat an infection, labelled 1 and 2 (not A and B). The vector $\mathbf{x}$ is populated by setting $\mathbf{x} = (S, R_1, R_2, X)$ where S denotes the proportion of patients in the hospital infected by a drug-susceptible pathogen. The variable R_1 then represents the proportion of patients infected by a drug-1-resistant pathogen, similarly for R_2, and then X is the proportion of uncolonised patients. The dynamical system, then, is this:

$$\frac{dS}{dt} = \mu(m - S) - (\tau_1 + \tau_2 + \gamma)S + \beta SX + \sigma\beta(c_1R_1 + c_2R_2)S, \tag{3a}$$

$$\frac{dR_1}{dt} = \mu(m_1 - R_1) - (\tau_2 + \gamma)R_1 + \beta(1 - c_1)R_1X - \sigma\beta(c_1S + (c_1 - c_2)R_2)R_1, \tag{3b}$$

$$\frac{dR_2}{dt} = \mu(m_2 - R_2) - (\tau_1 + \gamma)R_2 + \beta(1 - c_2)R_2X - \sigma\beta(c_2S + (c_2 - c_1)R_1)R_2, \tag{3c}$$

$$\frac{dX}{dt} = \mu(1 - m - m_1 - m_2 - X) + ...$$

$$... + (\tau_1 + \tau_2 + \gamma)S + (\tau_2 + \gamma)R_1 + (\tau_1 + \gamma)R_2 - \beta X(S + (1 - c_1)R_1 + (1 - c_2)R_2). \tag{3d}$$

It contains several parameters, μ, σ, m, m_1, m_2, γ, β, α, $\tau_{\max}$, c_1 and c_2, the meanings of which are given in the following table (taken from the main text).

Table 1 – The meaning of the parameters in equation (3).

parameter	meaning
m, m_1, m_2	Patients enter hospital in states S, R_1 and R_2 at rates $\mu m, \mu m_1$ and μm_2
τ_1, τ_2	Rate of use of drugs 1 and 2 per unit time (days)
c_1, c_2	Fitness cost of resistance to pathogens
σ	Relative rate of secondary colonization to primary colonization
β	Rate constant for colonization of uncolonized individuals
μ	Rate of patient turnover in the hospital
α	Represents physician compliance with cycling program
γ	Untreated patients colonized by susceptibles remain colonized $1/\gamma$ days on average

Since τ_1 and τ_2 represent the rates of applying drugs 1 and 2 and as everyone is treated with one of the drugs, it follows that $\tau_1 + \tau_2 = \tau$, the latter being a fixed constant. The

antibiotic stewardship problem from [3], stated as an optimal control problem, is then to seek a function $\tau_1(t)$ so that the performance, namely the proportion of all infected patient days due to drug-resistant infection, i.e.

$$\int_0^T R_1 + R_2 \; dt,$$

is minimised, where T denotes some fixed length of time for which the system is observed.

Even subtle changes in model parameters can be important. For example, the above question in optimal control is likely to have a different optimal solutions for each value of T, although this control is a difficult object to determine for these equations and so demonstrating this idea numerically is not straightforward.

Now, the second exemplar uses a slightly different terminological convention [5] with 'A' and 'B' for the drug labels and it sets $\mathbf{x} = (\mathsf{x}, \mathsf{y_w}, \mathsf{y_a}, \mathsf{y_b})$. Here x is the density of patients uninfected by a pathogen. The quantity $\mathsf{y_w}$ represents the number of patients infected by a wild-type pathogen strain, $\mathsf{y_a}$ denotes the number of patients with a drug-A resistant strain, similarly for drug-B resistant $\mathsf{y_b}$. The model is the following [5, Case III]:

$$\frac{d}{dt}\mathsf{x} = \lambda - d\mathsf{x} - b(\mathsf{y_w} + \mathsf{y_a} + \mathsf{y_b})x + r_w\mathsf{y_w} + r_a\mathsf{y_a} + r_b\mathsf{y_b} + ... \quad \text{(4a)}$$
$$... + h(1-s)((f_a + f_b)\mathsf{y_w} + f_a\mathsf{y_b} + f_b\mathsf{y_a}),$$

$$\frac{d}{dt}\mathsf{y_w} = (b\mathsf{x} - c - r_w - h(f_a + f_b))\mathsf{y_w}, \quad \text{(4b)}$$

$$\frac{d}{dt}\mathsf{y_a} = (b\mathsf{x} - c - r_a - hf_b)\mathsf{y_a} + hsf_a\mathsf{y_w}, \quad \text{(4c)}$$

$$\frac{d}{dt}\mathsf{y_b} = (b\mathsf{x} - c - r_b - hf_a)\mathsf{y_b} + hsf_b\mathsf{y_w}, \quad \text{(4d)}$$

where the epidemiological parameters are $\lambda,\ d,\ c,\ h,\ r_w,\ s,\ r_a,\ r_b$ and b whose interpretation is contained in Table 2. There are no multidrug-resistant strains in (4) and although that case has been considered in [5], for brevity we do not discuss it.

The purpose of applying (4) to the problem of antibiotic deployment is entirely analogous to (3). Given that f_a and f_b represent the fraction of the population treated with drugs A and B and assuming everyone is treated, so that $f_a + f_b = 1$, we then seek a function $f_a(t)$ so that the total of all patient infected-days, i.e.

$$\int_0^T \mathsf{y_w} + \mathsf{y_a} + \mathsf{y_b} \; dt,$$

is minimised. This too is a question in optimal control (whose solutions we do not compute).

4. Generic mathematical structure: the optimality problem

Solutions of (1), $\mathbf{x}$, depend nonlinearly on the protocol A and to make this explicit we write the solution as $\mathbf{x}(t; A)$. We can then determine the 'performance', $\mathcal{P}$, of a protocol, $A(t)$, by taking a weighted sum along components of the vector $\mathbf{x}$, setting

$$\mathcal{P}(A) := \int_0^T (\mathbf{x}(t; A), \mathbf{w})dt,$$

Table 2 – The meaning of the parameters in equation (4).

parameter	meaning
f_a, f_b	the fraction of patients treated with antibiotic A and B
r_w, r_a, r_b	recovery rates of wild-type, A-res and B-res infected hosts
b	transmission rate of infection
h	maximum rate at which patients are treated
s	fraction of patients that acquire resistance when treated
d	per capita death rate of uninfected hosts
λ	arrival rate of uninfected hosts
c	infected hosts' death rate

where $\mathbf{w} \geq \mathbf{0}$ is a weight vector and $(\cdot, \cdot)$ denotes Euclidean inner product. $\mathcal{P}$ also depends on the value of $\mathbf{x}(0)$ but we have suppressed this dependence in the notation for brevity. Just as the two exemplars above have done, different choices can be made for $\mathcal{P}$. For example, if $\mathbf{w} = (0, 1, 1, 0)$ then $\mathcal{P}$ is a measure of drug-resistant infection in (3). Or we could set $\mathbf{w} = (0, 1, 1, 1)$ where $\mathcal{P}$ then represents the totality of infected patient days in (4).

Ideally, we would like to solve the optimality problem of determining functions A that achieve the best performance

$$\min\{\mathcal{P}(A) : A \text{ in some antibiotic control strategy space}\}$$

and the worst performance

$$\max\{\mathcal{P}(A) : A \text{ in the same antibiotic control strategy space}\}$$

where the space of functions for A would have some clinical relevance. Bounded measurable functions in $L^\infty([0, T], [0, 1])$ is one choice that could be used to show that controls exist [1, 2] for these optimisation problems. However, it is not clear that this space is necessarily clinically relevant because it contains many more strategies than a hospital could ever realistically implement, which includes so-called chattering controls or oscillatory controls that attempt to exchange drugs every minute. Nevertheless, an optimal control does exist in that particular space under mild mathematical restrictions on the models.

Now, the set of all cycling protocols will be written Cyc where a protocol $t \mapsto A_{\omega,\varphi}(t)$ in Cyc is a periodic, measurable function of period ω, restricted to the interval $[0, T]$ taking only the values 0 or 1 for each t and φ is a phase shift parameter. So, if $\mathcal{A}_\lambda$ is a 1-periodic function on the real line taking the value 1 on $[0, \lambda]$ and the value 0 on $[\lambda, 1]$, where $0 < \lambda < 1$, and if $A_{\lambda,\omega,\varphi}(t) = \mathcal{A}_\lambda(\omega t - \varphi)$ where $\omega > 0$ and $\varphi > 0$ are arbitrary, this is one way of forming elements of Cyc which bias the time spent giving one drug over the other according to the value of λ. A function that biases antibiotic choice towards the other drug is, then,

$$A_{\lambda,\omega,\varphi}(t) = 1 - \mathcal{A}_\lambda(\omega t - \varphi).$$

The set of all mixing protocols, Mix, is simpler. It is the set of constant functions taking any value ν between 0 and 1. The performance distribution for the cycling protocols is then

$$\{\mathcal{P}(A) : A \in \mathsf{Cyc}\} \text{ and } \{\mathcal{P}(\nu) : \nu \in \mathsf{Mix}\}$$

in the case of mixing.

It has been proven [1] that cycling and mixing performance distributions necessarily overlap in the manner illustrated in Figure 3 in the main text. This is the idea that, given a mathematical model with fixed parameters, there is a family of mixing protocols (parameterised by ν) and a family of cycling protocols (parameterised by $(\lambda, \omega, \varphi)$) and every protocol from within these families can be evaluated according to the performance functional, $\mathcal{P}$. If one forms the distribution of all performances across the entire family of mixing and cycling protocols, these two distributions are structured such that the support of the cycling distribution contains the support of the mixing distribution [1]. Thus, if one picks a mixing protocol at random, say a sub-optimal mixing, with probability one there is a cycling that performs better than this mixing, but there is also a cycling that performs worse. This is why Figure 6 of the main text cannot arise.

There is the following caveat to this result: it is possible that optimal mixing has the same performance as the most rapidly cycling protocols and that no cycling can better optimal mixing [2, 1]. However, even then, Figure 3 from the main text will still apply but with the minor modification that the support of the cycling and mixing performance distributions 'touch' each other at their extremes. The following section sets this statement in mathematical language.

It is possible that optimal mixing outperforms all the cyclings so that, for any $a \in \mathsf{Cyc}$, there results

$$\mathcal{P}(a) > \min\{\mathcal{P}(\nu) : \nu \in \mathsf{Mix}\}.$$

However, since constant functions, like ν here, can be approximated in weak, or weak*, topologies of Lebesgue spaces by measurable functions inside Cyc, one can find sequences a_n, with $a_n \in \mathsf{Cyc}$, such that

$$\mathcal{P}(a_n) \to \min\{\mathcal{P}(\nu) : \nu \in \mathsf{Mix}\}$$

as $n \to \infty$ because there is a (weak* Banach-space) topology with respect to which the limit $a_n \to \nu$ is satisfied. This idea [1] is the mathematical basis of the next section.

4.1. **Best-case, worst-case inequalities.** Under a restriction called 'control dissipativity', the following result is proven in [2, 1] on the nature of $\{\mathcal{P}(A) : A \in \mathsf{Cyc}\}$ and $\{\mathcal{P}(\nu) : \nu \in \mathsf{Mix}\}$. The best performance in the set of antibiotic cycling protocols is at least as good as the best performance of the mixings:

$$\overbrace{\inf\{\mathcal{P}(A) : A \in \mathsf{Cyc}\}}^{\text{optimal cycling performance}} \leq \overbrace{\min\{\mathcal{P}(\nu) : \nu \in \mathsf{Mix}\}}^{\text{optimal mixing performance}} . \tag{5}$$

However, the worst possible performance of the antibiotic cycling protocols is at least as bad as the worst of the mixings:

$$\overbrace{\sup\{\mathcal{P}(A) : A \in \mathsf{Cyc}\}}^{\text{worst cycling performance}} \geq \overbrace{\max\{\mathcal{P}(\nu) : \nu \in \mathsf{Mix}\}}^{\text{worst mixing performance}} . \tag{6}$$

Inequalities (5) and (6) are entirely analogous to the analysis of the toy scenario from the main text and justify Figure 3, also in the main text*.

*Technical comment: sup and inf in inequalities (5) and (6) replace the properties of having a max and a min, respectively, in the case where a protocol family has a sequence of near-optimally performing cycling

We note that (5) and (6) can fail if the performance function $\mathcal{P}$ is not formed from a mathematical structure which is continuous with respect to weak or weak* convergence in Lebesgue spaces. However, no mathematical model in the literature that we are aware of fails to have these properties and the following are sufficient for this: a control affine structure with respect to $A(t)$, like (2), the linearity of $\mathcal{P}$ with respect to $\mathbf{x}$ and (2) having *a priori* bounded solutions whenever $A(t)$ is an almost everywhere bounded, measurable function.

5. Random mixing

Random mixing refers to the case whereby $A(t) \equiv 1/2$ in (1) whereas *optimal mixing* is the constant value of $A(t) \equiv \nu$ between zero and one for which the minimum, ν, is achieved in (5). The *worst mixing* is the constant value of A for which the maximum, ν, is achieved in (6).

It is clear that the mathematical models of the form (2) for which random mixing (aka maximal spatiotemporal heterogeneity) is the solution of the optimal stewardship problem must require a number of 'symmetries' in their model structure. This must be the case because, for mixing to be optimal, constant optimal controls are asking a dynamical model, with its changing behavioural dynamics, to have an optimal control that never changes. Mathematical models that satisfy this property must obey certain symmetries, as we now show.

To see this, one can argue from a perspective of elementary calculus that, if we go in search of optimal mixing, $\mathcal{P}(\nu)$ has a maximum in the space of all mixing functions at $\nu_* = 1/2$ when

$$\frac{d}{d\nu}\mathcal{P}(\nu_*) = 0.$$

This condition can be rewritten in the form

$$\frac{d}{d\nu}\int_0^T (\mathbf{x}(t;\nu), \mathbf{w})dt = 0,$$

or, assuming certain technicalities are satisfied,

$$\int_0^T \left(\frac{d\mathbf{x}}{d\nu}(t;\nu), \mathbf{w}\right) dt = 0. \tag{7}$$

The latter condition is a symmetry which shows that the weight vector $\mathbf{w}$ must be orthogonal to the variation of the state, $\mathbf{x}$, with respect to the control variable ν if random mixing is to be optimal. It follows that random mixing cannot be optimal in mathematical models for which (7) cannot be satisfied.

We can compute one such symmetry by noting that $\frac{d\mathbf{x}}{d\nu}(t;\nu) = \mathbf{y}$ satisfies an adjoint equation, namely

$$\frac{d}{dt}\mathbf{y} = \nabla F(\mathbf{x})\mathbf{y} + \nu_* \nabla G(\mathbf{x})\mathbf{y} + G(\mathbf{x}), \tag{8}$$

which shows that (7) is placing restrictions on the nature of the functions F and G in order for mixing to be optimal. Moreover, it can be shown (see [2] where these optimality calculations

---

strategies that does not converge to an optimal cycling strategy. This observation is needed as while there does exist an optimal strategy and an optimal mixing strategy, there need not exist an optimal cycling strategy.

are carried out in detail) that there are infinitely many analogous symmetries that must hold for random mixing to be optimal. These symmetry arguments, like the one above, indicate that there is nothing particularly special about random mixing, so it must also be the case that symmetries like (7) apply in order that any mixing solution (a constant ν_* between 0 and 1) be optimal.

There is a more direct way to see what conditions might need to be satisfied in order for mixing not to be optimal. For example, if it were the case that

$$\frac{d}{d\nu}\int_0^T (\mathbf{x}(t;\nu),\mathbf{w})dt > 0,$$

or

$$\frac{d}{d\nu}\int_0^T (\mathbf{x}(t;\nu),\mathbf{w})dt < 0,$$

for all $\nu \in (0,1)$, then the hospital-wide use of just one antibiotic will be better than mixing. In practise this condition could apply to a particular model scenario when resistance to one of the drug has fixed in the pathogen population so that patients can only be treated with the second drug. It is clear that random mixing, or indeed any mixing, cannot be the optimal way of treating patients in this particular situation for many choices of performance measure.

6. Optimal Control Computations

We can seek to show more directly that mixing is not the optimal behavioural strategy by computing the strategy that is optimal and there are tools that allow this to be done. Indeed, one can use these tools to determine the optimal antibiotic usage strategy for (3) from the main text which is a model taken from [14] and slightly modified. The tools alluded to here fall within the framework of numerical control theory and algorithms to solve these problems are applicable to equation (3) from the main text [12].

6.1. **Optimal Control Formulation.**

In order to determine these solutions we present the following background. First, the approach of [14] states the following. Given two antibiotics (denoted 1 and 2), the model assumes patients are partitioned according to their response to treatment: patient class 1 consists of patients infected with bacteria resistant to antibiotic 2, and vice versa. $I_j(t)$, for $j = 1, 2$, then denotes the abundance of patients infected with bacteria resistant to antibiotic $3 - j$, at time $t \geq 0$.

A control input $a(t)$ is the fraction of patients treated with antibiotic 1 (at time t), while $1 - a(t)$ is the fraction of patients treated with antibiotic 2 so that $a(t)$ takes values only in the interval $[0, 1]$. Generalizing the model of [14], we allow the patient groups to have different admittance and clearance rates:

$$\begin{cases} \dot{I}_1 = m_1 + I_1(1 - I_1 - I_2) - \gamma_1 a(t) I_1, \\ \dot{I}_2 = m_2 + I_2(1 - I_1 - I_2) - \gamma_2 (1 - a(t)) I_2. \end{cases} \tag{9}$$

Here, m_j is the admittance rate of patients of the j-th group and γ_j is the clearance rate when patients in the j-th group receive an effective treatment.

Given a time $T \geq 0$, our goal is to determine a strategy that minimises the total infection over the observation time window $[0, T]$. To achieve this we introduce the performance

functional $P : \mathbb{R}^2 \to \mathbb{R}$

$$P(a) = \int_0^T I_1(\tau) + I_2(\tau) d\tau; \tag{10}$$

note that the dependence of P on a is made through I_1 and I_2 which themselves depend on a in (9). Note also that P depends on the two-dimensional vector $(I_1(0), I_2(0))$ which is the initial state of the system. In the remainder of this discussion, any two-dimensional vector (I_1, I_2) will be written as just 'I', we will also write $P(a, I)$ for P.

Seeking the control a^+ of (9) that minimises total infection, P, we follow the standard method for solving this optimality problem which introduces the 'cost to go' performance functional:

$$P_t(a, I) := \int_t^T I_1(\tau) + I_2(\tau) d\tau.$$

We now introduce the *value function*: this function $\varphi(I, t)$ is the best (lowest) possible performance observed if we start the control problem at a value $t \in [0, T]$ from the state point $I \in \mathbb{R}^2$, with that performance taken over all possible controls $a : [t, T] \to [0, 1]$. More formally,

$$\varphi(I, t) = \inf_{a \in L^\infty([t,T],[0,1])} P_t(a, I). \tag{11}$$

Notice that the value function satisfies a boundary condition $\varphi(I, T) = 0,\ \forall I \in \mathbb{R}^2$. Now, φ satisfies a nonlinear partial differential equation called the *Hamilton-Jacobi-Bellman* equation that we can state if we first define the model in the form of a function

$$f(I, \alpha) := (m_1 + I_1(1 - I_1 - I_2) - \gamma_1 \alpha I_1, m_2 + I_2(1 - I_1 - I_2) - \gamma_2(1 - \alpha)I_2).$$

Theorem 6.1 (HJB)**.** *Assume that the value function φ is a C^1 function of the variables (I, t). Let $H : \mathbb{R}^2 \times \mathbb{R}^2 \to \mathbb{R}$ be the function*

$$H(I, p) := \min_{\alpha \in [0,1]} \Big\{ (f(I, \alpha), p) + I_1 + I_2 \Big\}, \tag{12}$$

for $I, p \in \mathbb{R}^2$. Then φ solves the following nonlinear partial differential equation for $I \in \mathbb{R}^2$ and $t \in [0, T)$:

$$\frac{\partial \varphi}{\partial t}(I, t) + H\big(I, \nabla_I \varphi(I, t)\big) = 0, \tag{HJB}$$

with terminal condition

$$\varphi(I, T) = 0, \quad \forall I \in \mathbb{R}^2. \tag{13}$$

Thanks to theorem HJB, the optimal control, a^+, can be obtained in two steps: 1) determine φ by solving equation (HJB) with condition (13); 2) For each $I \in \mathbb{R}^2$, $t \in [0, T]$ define $a^*(I, t) \in [0, 1]$ to be the value where the minimum in (HJB) is attained, meaning $a^*(I, t)$ solves the equation

$$\frac{\partial \varphi}{\partial t}(I, t) + f(I, a^*(I, t)) \cdot \nabla_I \varphi(I, t) + I_1(t) + I_2(t) = 0.$$

Then solve the differential equation

$$\frac{d}{ds}\mathbf{I} = f(\mathbf{I}, a^*(\mathbf{I}, s)), \qquad \mathbf{I}(t) = I,$$

whereafter one has $a^+(t) = a^*(\mathbf{I}(t), t)$. An introduction to the theory of optimal control that explains this reasoning in more detail can be found here:

`math.berkeley.edu/~evans/control.course.pdf`

6.2. **Optimal controls for** [14]**.** Following the above approach, we computed the value function φ and then the optimal control a^+ for (9), determining the function φ by solving (HJB) numerically using a MATLAB toolbox [12]. Some necessary details of that implementation are given in section 6.2.1 below.

Now, H in (12) associated with problem (9) is given by:

(14)

$$H(I,(p_1,p_2)) = \min_{\alpha\in A}\Big\{\sum_{k=1}^{2} p_k\left[m_k + I_k(1-I_1-I_2)\right] - p_1\gamma_1\alpha I_1 - p_2\gamma_2(1-\alpha)I_2 + I_1 + I_2\Big\}$$
$$= I_1 + I_2 - p_2\gamma_2 I_2 + \sum_{k=1}^{2} p_k\left[m_k + I_k(1-I_1-I_2)\right] + \min_{\alpha\in[0,1]} \alpha\left(p_2\gamma_2 I_2 - p_1\gamma_1 I_1\right).$$

From here, assuming for the moment that we have determined φ, it is possible to express a^* as a function of I, and t:

$$a^*(I,t) = \begin{cases} 1 : & \gamma_2 I_2 \frac{\partial\varphi(I,t)}{\partial I_2} < \gamma_1 I_1 \frac{\partial\varphi(I,t)}{\partial I_1}, \\ 0 : & \text{otherwise.} \end{cases}$$

Do note the formal resemblance between the definition of a^* and equations (1) and (2) in the main text that describes the optimal strategy in the toy model.

6.2.1. *Additional information on MATLAB implementation.* Since the MATLAB toolbox [12] is designed to solve (HJB) equations with respect to value functions φ with initial conditions of the form $\varphi(I,0) = g(I)$, for $I \in \mathbb{R}^2$ and for some $g : \mathbb{R}^2 \to \mathbb{R}$, we had to reformulate our terminal condition problem accordingly, this was accomplished with a change of variable $t \leftarrow T - t$. More formally, let us define $\tilde{\varphi}(I,t) := \varphi(I, T-t)$, where φ is the value function solving (HJB) with terminal condition (13). It is clear that, for all $I \in \mathbb{R}^2$,

$$(15)\quad \tilde{\varphi}(I,0) = 0, \quad \frac{\partial\tilde{\varphi}}{\partial t}(I,t) = -\frac{\partial\varphi}{\partial t}(I,T-t), \quad \nabla_I\tilde{\varphi}(I,t) = \nabla_I\varphi(I,T-t), \quad \forall t \in (0,T].$$

After rewriting (HJB) in the following form

$$\frac{\partial\varphi}{\partial t}(I,T-t) + H\big(I, \nabla_I\varphi(I,T-t)\big) = 0, \qquad \forall t \in (0,T],$$

we can use (15) to reformulate (HJB) as an initial value problem with respect to $\tilde{\varphi}$:

$$\frac{\partial\tilde{\varphi}}{\partial t}(I,t) - H\big(I, \nabla_I\tilde{\varphi}(I,t)\big) = 0, \qquad \forall t \in (0,T],$$

with initial condition $\tilde{\varphi}(I,0) = 0$, $\forall I \in \mathbb{R}^2$. Once we numerically solve this problem with respect to $\tilde{\varphi}$, we can obtain the numerical solution of φ from the definition of $\tilde{\varphi}$.

The MATLAB toolbox also requires an additional input: a function β expressing upper bounds on the absolute values of $\frac{\partial H}{\partial p_1}$ and $\frac{\partial H}{\partial p_2}$. More precisely, given the set

$$P := [p_1^{\min}, p_1^{\max}] \times [p_2^{\min}, p_2^{\max}] \subseteq \mathbb{R}^2,$$

where $p_i^{\min} < p_i^{\max}$ for $k = 1, 2$, the following function $\beta : \mathbb{R}^2 \to \mathbb{R}_+$ is required:

$$\beta_k(I) := \max_{p \in P} \left| \frac{\partial H}{\partial p_k}(I, p) \right|, \qquad k = 1, 2.$$

To obtain estimates of β_1 we first need the partial derivative $\frac{\partial H}{\partial p_1}$. The function H in (14) can be rewritten as

$$H(I,p) = \begin{cases} \sum_{k=1}^{2} I_k + p_k \left[m_k + I_k(1 - I_1 - I_2)\right] - p_1\gamma_1 I_1, & \gamma_2 I_2 p_2 \le \gamma_1 I_1 p_1, \\ \sum_{k=1}^{2} I_k + p_k \left[m_k + I_k(1 - I_1 - I_2)\right] - p_2\gamma_2 I_2, & \gamma_2 I_2 p_2 \ge \gamma_1 I_1 p_1, \end{cases} \tag{16}$$

and we note that if p satisfies $\gamma_2 I_2 p_2 = \gamma_1 I_1 p_1$, then $H(I,p)$ can be expressed in two ways. From (16) we compute the partial derivative of H with respect to p_1:

$$\frac{\partial H}{\partial p_1}(I,p) = \begin{cases} m_1 + I_1(1 - I_1 - I_2) - \gamma_1 I_1, & \gamma_2 I_2 p_2 \le \gamma_1 I_1 p_1, \quad \text{(17a)} \\ m_1 + I_1(1 - I_1 - I_2), & \text{otherwise.} \quad \text{(17b)} \end{cases}$$

To express β_1 as a function of I, we focus on the subset of P for which the formula in (17a) holds, that is $Q(I) := \{p \in P \mid \gamma_2 I_2 p_2 \le \gamma_1 I_1 p_1\}$. We observe that $\frac{\partial H}{\partial p_1}(I,p)$ is given by the formula in (17a) if and only if $Q(I) = P$. Moreover, being P the Cartesian product of two closed intervals $[p_1^{\min}, p_1^{\max}]$ and $[p_2^{\min}, p_2^{\max}]$, we have that $Q(I) = P$ if and only if $\gamma_2 I_2 p_2^{\max} \le \gamma_1 I_1 p_1^{\min}$. Similarly, $\frac{\partial H}{\partial p_1}(I,p)$ is always given by (17b) if and only if $Q(I) = \emptyset$, which holds if and only if $\gamma_2 I_2 p_2^{\min} > \gamma_1 I_1 p_1^{\max}$. Therefore, we can express β_1 as:

$$\beta_1(I) = \begin{cases} \left| m_1 + I_1(1 - I_1 - I_2) - \gamma_1 I_1 \right|, & \gamma_2 I_2 p_2^{\max} \le \gamma_1 I_1 p_1^{\min}, \\ \left| m_1 + I_1(1 - I_1 - I_2) \right|, & \gamma_2 I_2 p_2^{\min} > \gamma_1 I_1 p_1^{\max}, \\ \max\left(\left| m_1 + I_1(1 - I_1 - I_2) - \gamma_1 I_1 \right|, \left| m_1 + I_1(1 - I_1 - I_2) \right| \right), & \text{otherwise.} \end{cases}$$

A value for $\beta_2(I)$ can be obtained with an analogous approach, where the partial derivative of H with respect to p_2 is

$$\frac{\partial H}{\partial p_2}(I,p) = \begin{cases} m_2 + I_2(1 - I_1 - I_2), & \gamma_2 I_2 p_2 < \gamma_1 I_1 p_1, \\ m_2 + I_2(1 - I_1 - I_2) - \gamma_2 I_2, & \text{otherwise,} \end{cases}$$

and this leads to

$$\beta_2(I) = \begin{cases} \left| m_2 + I_2(1 - I_1 - I_2) \right|, & \gamma_2 I_2 p_2^{\max} < \gamma_1 I_1 p_1^{\min}, \\ \left| m_2 + I_2(1 - I_1 - I_2) - \gamma_2 I_2 \right|, & \gamma_2 I_2 p_2^{\min} \ge \gamma_1 I_1 p_1^{\max}, \\ \max\left(\left| m_2 + I_2(1 - I_1 - I_2) - \gamma_2 I_2 \right|, \left| m_2 + I_2(1 - I_1 - I_2) \right| \right), & \text{otherwise.} \end{cases}$$

Matlab codes used to solve the Hamilton-Jacobi-Bellman equations can downloaded here:

`http://www.cs.ubc.ca/~mitchell/ToolboxLS/`

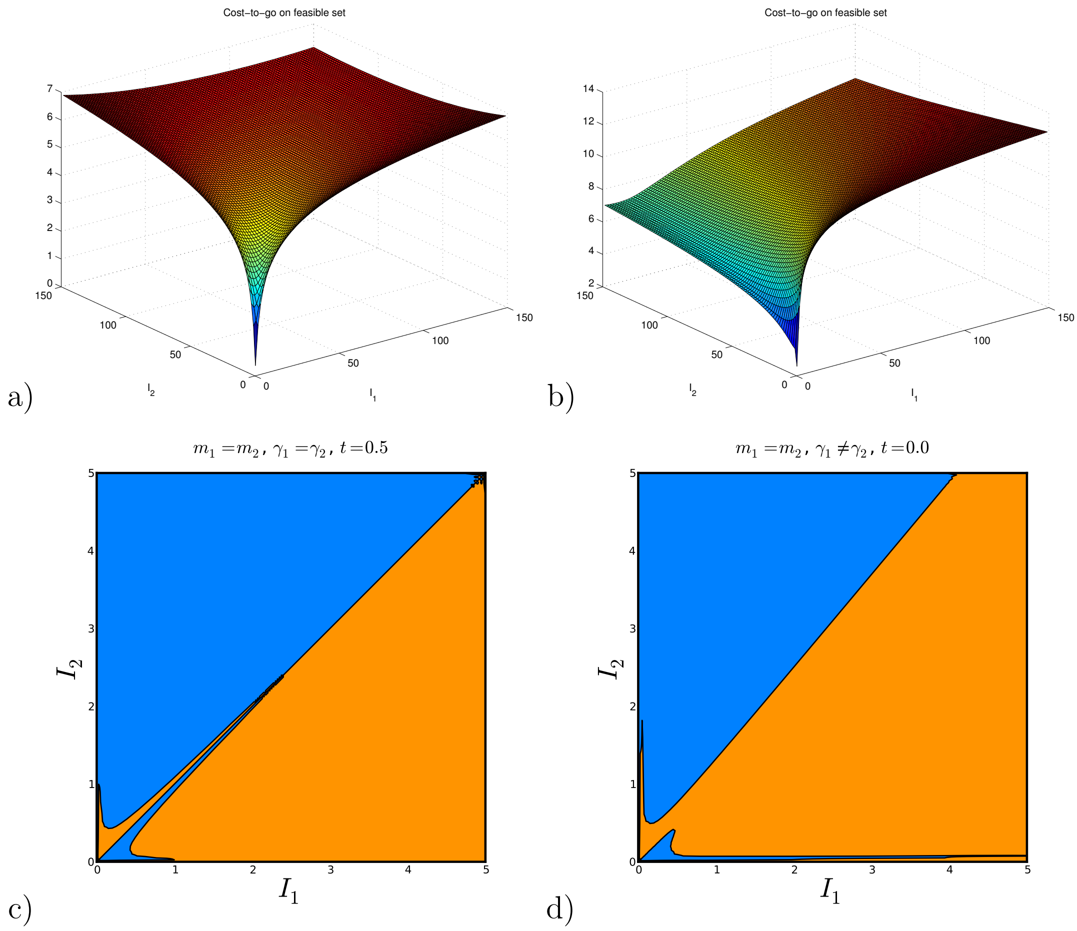

Supplement fig. 1 – Numerical solutions of the HJB equation associated with (9) in two different cases. One case where $\gamma_1 = \gamma_2$ (in a) and one where $\gamma_1 \neq \gamma_2$ (in b). Plots (c) and (d) show typical state-dependent optimal control decisions at different times, as determined from the value functions, $\varphi(I, t)$, shown in (a) and (b), respectively, where the blue and orange zones denote where one, or the other, drug should be used in the model at any given time. The lines at the boundary of the blue-orange zones indicate where drug switches should take place for the control to be optimal. As a result, the control laws derived from those solutions both state that only one drug should be used at a time and they should be exchanged when a certain ratio of I_1/I_2 is reached. Both drugs may only be used together when solutions lie on the common boundaries of the blue and orange regions. Note how those decisions are remarkably like the optimal solutions provided by the toy example in the main text (equations (1) and (2)).

6.3. **Numerically-computed optimal controls.** Supplement fig. 1 (note c) and d) in particular) shows optimal antibiotic stewardship decisions formulated as control laws that were obtained using the arguments of the previous section applied to equation (9). Supplement figs. 1(c) and (d) show that the rather more sophisticated approach provided by the theory of optimal control and equation (HJB) produce remarkably similar solutions to the toy arguments presented in the main text that lead to the derivation of equations (1) and (2) in that text.

These solutions are control laws which state that one should observe the dynamics of the system continually, which in practise means as often as possible. One should then assess the state of resistance in the population and treat everyone with the same drug, namely that drug which is appropriate for as many infections as possible. This is the drug which at the time of observation has the fewer resistant infections in the population. In other words, as individual cases cannot be inspected given the nature of the model (9), one should maximise the likelihood of giving an appropriate drug across the population treated.

We use this outcome to motivate the use of reactive (aka feedback) controls like

$$a_1(t) = \begin{cases} 1 & \text{if} \quad \mathsf{y_a}(t) < \mathsf{y_b}(t) \\ 0 & \text{if} \quad \mathsf{y_a}(t) > \mathsf{y_b}(t) \end{cases} \tag{18}$$

and

$$a_2(t) = \begin{cases} 1 & \text{if} \quad R_1(t) < R_2(t) \\ 0 & \text{if} \quad R_1(t) > R_2(t) \end{cases} \tag{19}$$

in the main text that we deployed in (3) and (4) after setting,

$$\tau_1(t) = \tau \cdot a_1(t), \;\; \tau_2(t) = \tau \cdot (1 - a_1(t))$$

in (3) and

$$f_a = a_2(t), \;\; f_b(t) = 1 - a_2(t)$$

in (4). We report the outcomes of the use of these controls in the main text, for instance in Figures 2 and 5.

7. Performance histograms from stochastic differential equation models

In order to explicitly account for random variability in clinicians' prescription patterns and to exploit this variability to produce performance histograms to illustrate how the performance inequalities (5) and (6) materialise in specific mathematical models, we now define stochastic versions of the model structure captured by (2). An introduction to the numerical solutions of stochastic differential equations [8] might be helpful for implementing these methods.

While the performance inequalities (5) and (6) have been established for the deterministic dynamical system (1), which includes (3) and (4) as special cases, continuous dependence properties of stochastic differential equations ensures that the inequalities (5) and (6) hold in an approximate sense for small-variance stochastic systems of the form discussed below.

So, now suppose that $\alpha(t)$ is a specified 'target' antibiotic deployment protocol that clinicians are expected to adhere to, whether mixing, cycling or something else, but suppose that it is not always possible for clinicians to adhere to this target. As a result, α is not implemented in mathematical models. To reflect this clinical variability we alter the protocol and let $A(t)$ represent a stochastically varying deployment protocol such that $\mathbb{E}(A(t)) = \alpha$ holds for each moment in time $t \geq 0$ where $\mathbb{E}(\cdot)$ denotes expectation. We therefore implement, instead, $A(t) = \alpha(t) + \sigma N(t)$ where α is the target protocol, σ^2 is the noise variance and $N(t)$ is a model of the noise with zero mean, unit variance.

If N can be posed as an element of a function space, starting from equation (2) and setting $A(t) = \alpha(t) + \sigma N(t)$, this rationale yields a differential equation of the form

$$\frac{d}{dt}\mathbf{x} = F(\mathbf{x}) + (\alpha(t) + \sigma N(t))G(\mathbf{x}). \tag{20}$$

A standard way of using noise in differential equations is to replace $N(t)$ with $dW(t)$ where $W(t)$ is a Wiener process and to produce performance data by simulating the following stochastic differential equation:

$$d\mathbf{x}(t) = (F(\mathbf{x}(t)) + \alpha(t)G(\mathbf{x}(t)))\,dt + \sigma G(\mathbf{x}(t))dW(t), \tag{21}$$

where $\mathbf{x}(0)$ is given. Here, α is a member of either Cyc or Mix.

Each numerical solution, $\mathbf{x}(t)$, of equation (21) produced by this method has its own performance and by simulating this model many times over we can form a performance histogram of an ensemble of targeted drug-usage strategies, $\alpha(t)$. When we repeat this procedure with $\alpha(t)$ taken to be different mixing and cycling protocols, we can produce approximate performance distributions for these two families of protocols.

This method is used to produce data for figures in the main text but this approach does have certain modelling drawbacks. For example, there is no reason the Euler-based, fixed-timestep numerical discretisations of (21) as described in [8] will maintain the positivity of solution components required by the fact that the models obey biological, physical and clinical situations which need positivity to make any sense. In addition, there is a non-zero probability that $\alpha(t) + \sigma N(t)$ can fall outside the interval $[0, 1]$ when $N = dW$ in those numerical solutions. If σ is small enough this will be a rare event, but it is also an undesirable artefact of this approach. The same can be said of the fixed, timestep Euler methods used to solve deterministic differential equations: they need not respect symmetries of the equations they discretise, like positivity, which is essential if our computational results are to be meaningful. In practise, problems like this meant that solutions of equations (21) were restricted to very small noise and small time steps so that these issues were never observed in practise in the 10^7 computational simulations effected per parameter set. However, had we sampled more solutions, these issues would inevitably have arisen.

So, to remedy some of them, we also implemented the following 'finite-dimensional' method for introducing noise which does not invoke standard stochastic calculus. This was done to ascertain whether any of the aforementioned problems materially affected our results. To implement this we first define a vector, $\boldsymbol{v} = (v_0, v_1, ..., v_n)$ of points where each $v_j \in [0, 1]$ is a random variable with defined mean m_j and variance σ_j^2 whose probability distribution, p_j, has support contained entirely in $[0, 1]$. We then define a mapping from $\mathbb{R}^{n+1}$ (where $\boldsymbol{v}$ lives) into the space of Lipschitz functions $C^{\mathrm{Lip}}([0, T], [0, 1])$ by requiring that $A = A(\boldsymbol{v})(t)$ is a piecewise linear function interpolating $\boldsymbol{v}$ at points $t = t_j = Tj/n$ for $j = 0, ..., n$. Clearly A and its weak derivative $\frac{d}{dt}A$ are elements of $L^\infty([0, T], [0, 1])$ and so the differential equation

$$\frac{d}{dt}\mathbf{x} = F(\mathbf{x}) + A(\boldsymbol{v})(t)G(\mathbf{x}). \tag{22}$$

is well-defined and even has C^2 solutions for each $\boldsymbol{v}$. Moreover, this structure allows us to exploit adaptive time-stepping algorithms that can be used to solve (22) and maintain, for

example, positivity of the numerical approximations of the solution $\mathbf{x}(t)$ whenever this constraint is applicable to the underlying differential equation (22). Moreover, useful properties such as the smooth dependence of $\mathbf{x}$ on $\boldsymbol{v}$ is immediate and so the performance inequalities (5) and (6) hold in an approximate sense if variance in this 'finite-dimensional noise' model is small. Note we make no attempt to take the limit $n \to \infty$ here because clinicians cannot make decisions on arbitrarily small timescales.

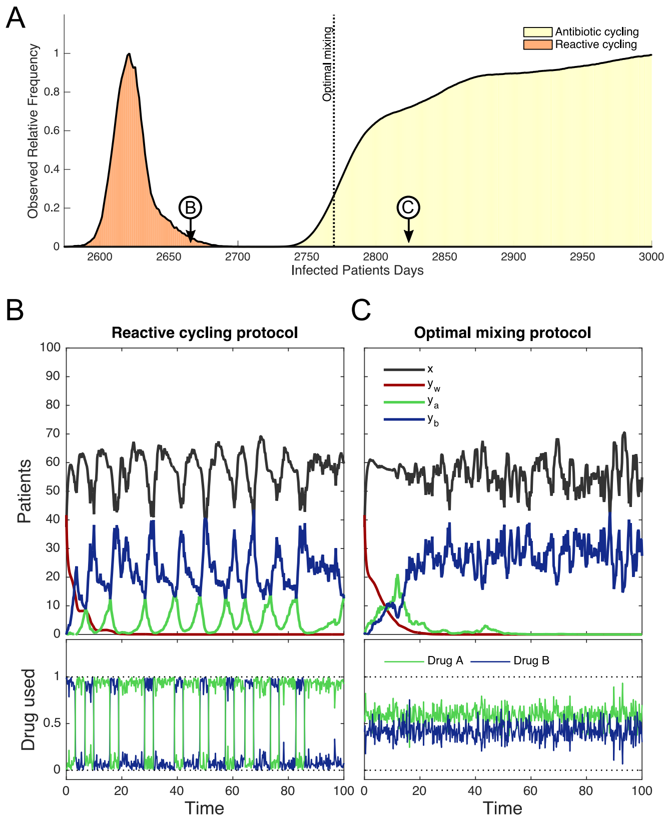

Supplement fig. 2 – This is the analogy of Figure 2 from the main text using 'finite dimensional' noise as defined in the text.

If we set $m_j = \alpha(t_j)$ then one can use (22) to generate performance histograms of the protocol α. To do this, for each value of $t = t_j$ we defined σ_j to be the same value, however, we allowed the support of the probability distribution, p_j, in practice the uniform distribution, to vary at each timepoint, t_j, so that the probability distribution for the antibiotic control has support contained entirely inside the interval $[0, 1]$ at all times. We did this to prevent different variances arising in the values of $A(t)$ at different times which could happen when, for instance, $\alpha(t)$ takes on, or is close to, one of the values 0 or 1 at the boundary of the control interval $[0, 1]$.

When we implemented this methodology in Matlab, we found broad agreement between it and the more common approach that uses a Wiener process to represent noise that was used to produce data for the figures in the main text. This is borne out by Supplement figs. 2, 3 and 4 which show close resemblance to the equivalent figures in the main text.

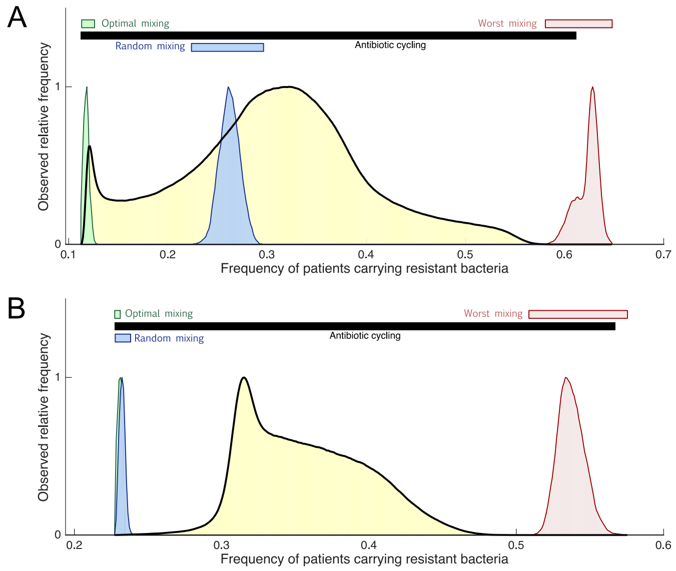

Supplement fig. 3 – The analogy of Fig. 4 from the main text using 'finite dimensional' noise.

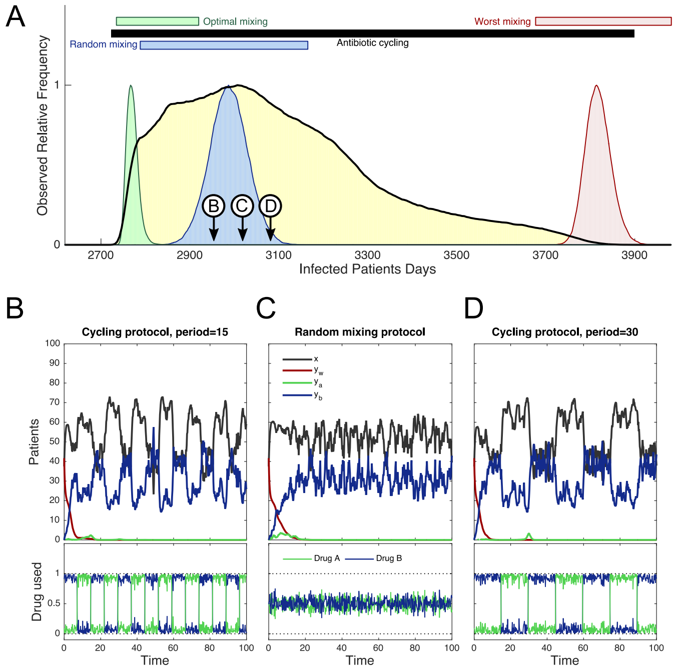

Supplement fig. 4 – The analogy of Fig. 5 from the main text using 'finite dimensional' noise.

NB$_1$. A horizontal bar is used in the above figures and in the main text to denote the support of the entire empirical dataset. So, if $\mathcal{D}$ is that dataset, a horizontal 'bar' is drawn from $\min(\mathcal{D})$ to $\max(\mathcal{D})$ to indicate that support. As all histograms that use stochastic simulations in this paper are based on at least 10^4 numerical simulations, we can use t-tests and anovas to obtain p-values less than 10^{-15} to support any claim that two datasets for which the bars do not overlap come from distributions with different means. Importantly,

all such bars for the cycling protocols in all the figures overlap with bars from the mixing protocols. This outcome is consistent with the schematic presented in Figure 3 in the main text.

NB$_2$. Cycling protocols were implemented using the following Matlab-style pseudocode that we used to decide when to switch from the use of one drug to another:

```
for first = 0:1
    % use drug 1 first, then drug 2 on a second pass
    parameters.useDrugOneFirst = first;
    for k = 1:repeats
        % the first switch no later than T/k, here uniform dist-d in [0,T/k]
        taus1 = rand(1,simulations)*T/k;
        % as the sims are repeated, the first switch gets progressively earlier
        % the second switch no later than T
        taus2 = taus1 + (T-taus1).*rand(1,simulations)/k;
        for j = 1:simulations
            % define antibiotic deployment on [0,T] from switch times
            % A is zero or one, switching values at these times
            A = makeSwitches([taus1(j) taus2(j)],T);
            % get a differential equation model trajectory using A
            traj = OneCyclingTrajectory(parameters,A);
            % get the performance and store it
            Payoffs = [Payoffs getPerformance(parameters, traj)];
        end
    end
end
```

We also tested switching times using different sampling distributions on the $[0, T]$ interval. In particular we used a (non-random) uniform spacing in addition to the uniform and Poisson distributions to produce the switching times. The shape of the resulting payoff distribution does change as this distribution alters, but not its overlap with respect to the range of performances of the family of mixing distributions. We also allowed the numbers zero and one to vary in the above pseudocode so that when a mixing protocol of mean value β and variance σ^2 was compared against a cycling protocol, the cycling protocol also had variance σ^2 in the control values it took, while constraining those values to lie in the interval $[0, 1]$.

8. Individual-based model: a stochastic, population-genetic model of within-host treatments

We also implemented an 'agent-based' modelling framework to test different antibiotic deployment protocols whereby patients are considered to be individual hosts colonised by a commensal bacterium and infected by a rapidly evolving pathogen. In order to clear pathogens from the host, patients are treated with a continuous dosing regimen (akin to an intravenous supply) with one of two antibiotics found in the 'patient' at concentrations that hereafter we denote using variables $A(t)$ and $B(t)$. The treatment for each patient is determined by functions $\alpha(t)$ and $\beta(t)$, each of these denoting the input rate of antibiotic to the patient host. Both functions are bounded, $0 \leq \alpha(t) \leq A_0$ and $0 \leq \beta(t) \leq B_0$, where A_0 and B_0 denote the maximum dose permitted for each drug in the intravenous supply.

In the following, the variable $C(t)$ will represent the density of commensal bacteria in the host and

$$\boldsymbol{P}(t) = (P_s(t), P_a(t), P_b(t), P_{ab}(t))$$

is a vector containing the densities of different drug-resistant, pathogenic mutants, where P_s represents a drug-susceptible wild-type pathogen. The entries P_a and P_b represent pathogens resistant to antibiotics A and B respectively and P_{ab} is a multidrug-resistant mutant.

This model, although highly simplified, is based on the use of antibiotic drugs for which resistance can arise *de novo* through genetic mutations that alter the structure of a binding pocket targeted by the drug molecule [4]. For this reason, transition rates between different pathogenic bacterial genotypes due to mutations that confer drug resistance arise through a Markovian mutation process given by a matrix $\mathcal{M}_\epsilon$, where ϵ denotes a mutation rate. This mutation process is a near-identity stochastic matrix that can be decomposed into the form

$$\mathcal{M}_\epsilon = \begin{pmatrix} 1-\epsilon & \frac{1}{2}\epsilon & \frac{1}{2}\epsilon & 0 \\ \frac{1}{2}\epsilon & 1-\epsilon & 0 & \frac{1}{2}\epsilon \\ \frac{1}{2}\epsilon & 0 & 1-\epsilon & \frac{1}{2}\epsilon \\ 0 & \frac{1}{2}\epsilon & \frac{1}{2}\epsilon & 1-\epsilon \end{pmatrix} = (1-\epsilon)I + \epsilon \overbrace{\begin{pmatrix} 0 & \frac{1}{2} & \frac{1}{2} & 0 \\ \frac{1}{2} & 0 & 0 & \frac{1}{2} \\ \frac{1}{2} & 0 & 0 & \frac{1}{2} \\ 0 & \frac{1}{2} & \frac{1}{2} & 0 \end{pmatrix}}^{M}. \tag{23}$$

where I is the identity matrix, $0 < \epsilon \ll 1$ and M is a 4-by-4 matrix, $(m_{ij}) = M$, such that m_{ij} specifies the change in drug resistance profile due to each mutation. We neglect from this model the possibility that two simultaneous drug-resistance mutations can occur as this is a term of size $O(\epsilon^2)$ which is smaller than ϵ. Note, if $\mathbf{1}$ denotes a vector of ones, $\mathbf{1} = (1,1,1,1)^T$, then $\mathbf{1}^T\mathcal{M}_\epsilon = \mathbf{1}$ and $\mathbf{1}^T M = \mathbf{1}$ apply. Moreover, M is irreducible so that, for each (i,j), there is a number k depending on (i,j) such that the (i,j)-th entry of $\mathcal{M}_\epsilon^k$ is non-zero.

The per unit time growth rate of each bacterial pathogenic mutant is determined by the availability of a limiting carbon source that we denote by $S(t)$ and the concentration of antibiotics in the host act to reduce this growth rate. The susceptibility profile of each bacterial genotype (that we will distinguish below with a subscript asterisk placeholder $*$ that denotes each type) with respect to the concentration of both drugs is contained within the definition of a dimensionless inhibition function γ_*, where $0 \leq \gamma_*(A, B) \leq 1$. We model

the growth rate, G_*, of each mutant as a resource uptake function modulated by the inhibition coefficient, $\gamma_*(A,B)$, meaning

$$G_*(S,A,B) = u_*(S)\cdot\gamma_*(A,B). \tag{24}$$

Here $u_*(S)$ is a saturating Monod function with maximal growth rate μ_* and half-saturation constant K_*:

$$u_*(S) = \frac{\mu_*\, S}{K_* + S}. \tag{25}$$

Although we do not explicitly treat patients using antibiotic combinations, two drugs could be found inside the host simultaneously. For example if one has not cleared the host before the second is applied. We therefore gave the drugs a *synergistic* effect, assumed to be multiplicative with respect to single-drug inhibition:

$$\gamma_*(A,B) = \left(1 - \frac{\nu_*\, A}{\kappa_* + A}\right)\cdot\left(1 - \frac{\tilde{\nu_*}\, B}{\tilde{\kappa_*} + B}\right), \tag{26}$$

where κ_* and $\tilde{\kappa_*}$ represent the half saturation constants of the antibiotics A and B respectively while ν_* and $\tilde{\nu_*}$ control the level of maximal growth inhibition by each drug.

Each bacterial mutant has its own profile of drug susceptibility determined by the set of parameters $(\mu_*, \nu_*, \tilde{\nu_*}, \kappa_*, \tilde{\kappa_*})$. For instance, we say that P_a is resistant to drug A because $0 \le \gamma_a(A,\cdot) < \gamma_s(A,\cdot) \le 1$ when $A \gg 0$. Moreover, we assume a fitness (growth rate) cost of drug resistance whereby the resistant bacteria have lower growth rates than susceptible bacteria at low antibiotic concentrations. We therefore choose our parameters so that $G_s(S,A,B) > G_a(S,A,B)$ at low concentrations of A and, analogously, we impose a fitness cost of resistance for drug B.

Therefore, the vector $(S, C, \boldsymbol{P}, A, B)$ represents the state of each host in an evolutionary model for a population of commensal and pathogenic bacteria competing in a limited resource environment and subject to the effects of two antibiotics. The model itself is this:

$$\begin{aligned}
\tfrac{d}{dt}S &= d(S_0 - S) - c\cdot(u_c(S)C + u_s(S)P_s + u_a(S)P_a + u_b(S)P_b + u_{ab}(S)P_{ab}),\\
\tfrac{d}{dt}C &= G_c(S,A,B)C - dC,\\
\tfrac{d}{dt}\boldsymbol{P} &= \mathcal{M}_\epsilon(G(S,A,B)\otimes\boldsymbol{P}) - d\boldsymbol{P},\\
\tfrac{d}{dt}A &= \alpha(t) - dA - \beta_a\cdot A(C + P_s + P_a + P_b + P_{ab}),\\
\tfrac{d}{dt}B &= \beta(t) - dB - \beta_b\cdot B(C + P_s + P_a + P_b + P_{ab}),
\end{aligned} \tag{27}$$

with given initial conditions $\boldsymbol{x}(0) = (S_0, C_0, \boldsymbol{P}_0, A_0, B_0)$. The vector of growth rates, G, is defined by $G(S,A,B) = (G_s, G_a, G_b, G_{ab})$ and $\otimes$ here denotes the pointwise product of two vectors. Parameters β_a and β_b represent the binding rates of each bacterial phenotype to each of the corresponding antibiotic molecules, c denotes a resource-to-biomass conversion constant and d denotes the constant influx and efflux of resources from the host. Bacteria are also cleared from the system at rate d and drugs are washed out of the system at the same constant rate for both drugs. This model is therefore treating a chemostat as if it represented the dynamics of infection and treatment in a human, this idea clearly has many limitations and we explicitly discuss some of the limitations of our model later in this supplement.

Finally, the '*health state*' of a host is defined by the ratio of commensals and pathogens:

$$h(t) = \frac{C}{C + P_s + P_a + P_b + P_{ab}}, \tag{28}$$

and a host is deemed to have recovered from illness when h is above a prescribed threshold, given by the parameter $\delta > 0$.

9. A spatially explicit extension of the host model

We will now put patients into a representation of a hospital ward to mimic the spread of resistance and the impact treatment has on that dynamic. To do this we first defined a linear array of B beds indexed by the label $j \in \{1, 2, ..., B\}$. Now let $\mathbf{Q} = \{p_1, p_2, \ldots, p_n\}$ be a randomised queue of n patients, where each individual p_i is an initial condition for the above patient model whereby patients are assumed to be colonised by commensal and infected by a pathogenic mutant competing under the evolutionary dynamic described by the model (27). Our full ward model implements the diffusion of pathogens through the ward: every τ units of time, a proportion p of each bacterial mutant in each patient is transmitted to hosts of neighbouring beds.

When admitted to the ward, each patient host is assigned to an available bed where treatment will be given until the moment the patient is discharged, this occurs when the health state (28) is above the threshold δ. Now, if $t = T$ is the first time for which $h_i(t) = \delta$, where h_i is the health state of the i-th patient, we then say that the length of stay (LoS) of this patient is T units of time.

When one patient is discharged, the next from the queue is assigned to the available bed where it will also receive treatment until deemed healthy in this way. If a patient is admitted to the hospital and its initial health state is above the threshold, it is discharged immediately and the bed then becomes available. When every patient from the queue has been discharged from the ward, we compute the mean length of stay (MLoS) of this particular ordering of the queue and this particular treatment protocol.

There are two sources of stochasticity within our model: the initial frequency of resistance of each patient in the queue and the order of the queue itself. The former is a representation of community-acquired resistance and the latter is also relevant because changing the position of the patient in the queue can lead to a change in the treatment administered to that patient because of the context of the ward on admittance. This implies that by re-ordering the queue of patients and implementing the same drug usage strategy multiple times, we can obtain statistics on the LoS and so compare one antibiotic deployment strategy with another implemented on the same queue of patients.

There are clearly many different ways of assuming resistance is modelled in the patient queue due to the assumption we make on the prevalence of drug resistance in the community. We have, therefore, considered the effect that the levels of drug resistance in the community have on the efficacy of different hospital interventions by varying a parameter, $\rho \in [0, 1]$, that denotes the initial frequency of drug resistant alleles in an infected and queued patient, as illustrated in Supplement figs. 6 and 7. If $\rho = 0$ then every drug resistant pathogen in the system is nosocomial acquired, possibly via *de novo* mutation, while $\rho = 1$ denotes the case

where every patient in the queue is infected exclusively by drug-resistant pathogens even before admittance to the ward.

To describe these different community structures we use the term *addition scenario* to refer to the case where drug resistant pathogens coexist in the host alongside drug susceptible pathogens before treatment commences, as illustrated in Supplement fig. 6. By contrast, Supplement fig. 6 also shows a queue of patients generated under what we term a *replacement scenario*, so-called because patients are infected exclusively by a single pathogenic phenotype, either resistant or susceptible, when treatment commences.

For the sake of brevity, simulations shown only implement the case where patients are infected under the addition scenario, our main conclusions are robust to some changes in the community model under consideration, but not all. The ward model as presented here and in the main text therefore shows that personalised strategies *can* outperform mixing and cycling, not that they *always* do under all circumstances. For instance, if all patients are infected with pathogens susceptible to either drug, a personalised strategy will not outperform mixing.

9.1. **Additional discussion of assumptions.** Let us summarise the nature of the ward model. First, it uses an individual patient infection model and the ward model that builds on that. The latter is a spatially-extended, individual-based population genetic model in which patients are explicit. Patients are admitted to the ward colonised by a commensal and infected in the community (which can spread in the ward) by an evolving pathogenic bacterium that compete for a limiting resource in a ecological and evolutionary dynamic. A patient is discharged from the hospital ward when pathogenic bacteria have been cleared from the system and commensals have re-colonised the host niche. The next individual from a randomised queue of patients is assigned to the newly-freed bed where treatment will be given until recovery.

For simulation purposes, our ward is a spatially explicit array of $B = 8$ beds with spatial and temporal dynamics described above. Different individualised and hospital-wide interventions are simulated until every patient from an ordered queue of $n = 100$ individuals is considered to be healthy. In order to obtain the statistical distributions on the mean length of stay (MLoS), the queue is re-ordered and the experiment repeated $N = 1000$ times.

The following are important assumptions used in the model:

- pathogens can evolve resistance to both of the two drugs due to point mutations and the accumulation of multiple mutations confers pathogens with multi-drug resistance;
- there is a fitness cost of drug resistance expressed by resistant pathogens having a lower growth rate than susceptible bacteria in the absence of antibiotic;
- there is no infection-induced mortality and, if left untreated, all patients will eventually recover, although more slowly than if treated;
- when located in the ward, patients are always treated with one of two possible drugs, the drug prescribed to each patient at any moment in time is decided based on the policy at the time of admittance;

The parameters considered in the simulations are given in Table 3 and the ward model was solved numerically using MATLAB (version 7.1) differential equation solvers and the Parallel Computing Toolbox.

9.2. **Modelling criticisms.** Many criticisms of the ward model are possible and this is a summary of just a few of these.

Q. Why is $C(t)$ not a vector containing the densities of many commensal species in, or on the host, all competing with the pathogen and on which evolution can act? A: it would be very difficult to realistically parameterise such a model, and what we present could be seen as an average over a potentially large community of bacteria. Note that we have ignored drug-resistance adaptation in the commensals but this will likely occur too.

Q. Why are we using a synergistic drug pair? A: this is often seen as the preferred interaction in clinical settings, but it is not key to our study. As no drug combinations are given, two drugs are only in the host because one of them did not clear from the previous treatment and we had to define *some* interaction. Do note that combinations are used in clinical practise so this is another case where our ward model, like other models in the literature, departs from reality.

Q. Why are we using this particular definition of health state and not, for example, just minimising pathogen density? A: there are many definitions we could choose, each with its own optimal strategy, we had to choose one so, somewhat arbitrarily, it was that one.

Q. These are chemostat models, in effect, so what does the dilution rate d mean for a patient? A: this is the expected time any cell stays inside the host, in effect a 'biomass turnover rate'. But indeed, there is no immune system in this model either, other than the commensal itself.

Q. What happens if a plasmid confers resistance by horizontal gene transfer? A: this is an important case we have neglected that is relevant for multi-drug resistance. We have considered the spatial transmission of resistant pathogens, but the explicit inclusion of a multi-resistance plasmid could change our results. However, none of the mathematical models that we discuss from the current literature on mixing and cycling have explicitly introduced plasmid-borne multi-resistance, either. This would be an important extension of the mixing and cycling model suite. One unpublished preprint that is available on the web, [7], does consider multi-drug resistance.

Table 3 – The microbiological parameters used for the individual-based model simulations. These values are not calibrated to reflect real PKPD of antibiotics inside patients, but they have been obtained from growth and inhibition experiments using *E.coli* and small molecule antibiotics that we do not describe here for the sake of brevity.

Parameter	Description	Value
N	Number of hospitals simulated (unless specified otherwise)	1000
n	Number of patients in the queue	100
B	Number of beds in hospital ward	8
p	Rate of bacterial transmission between neighbouring beds	$0.1/h$
δ	% of commensals level at which patients are discharged	90%
d	Rate of input and output of resources from each host	$0.4/h$
ϵ	Rate of point mutations in pathogens	0.01 per cell per divison
c	Resource-cell conversion rate in antibiotic-free environment	$10^8 cell/\mu g$
K	Bacterial half-saturation constant	$0.06\mu g/ml$
μ^*	Maximal resource uptake rate of bacterial type $*$ per hour (note $V^*_{max} = \mu^*/c$)	$\mu^c = 1.5/h$, $\mu^s = 1.3/h$, $\mu^a = 1/h$, $\mu^b = 1/h$, $\mu^{ab} = 0.87/h$
S_0	Resource supply concentration	$6\mu g/ml$
A_0	Antibiotic A maximum dose	$1\mu g/ml$
B_0	Antibiotic B maximum dose	$1\mu g/ml$
β_a	Antibiotic A cell binding rate	$1 \times 10^{-8}\mu g/cell/h$
β_b	Antibiotic B cell binding rate	$1 \times 10^{-8}\mu g/cell/h$
κ_1^*	Maximal growth inhibition of bacterial type $*$ by antibiotic A	$\kappa_1^c = 0.59ml/\mu g$, $\kappa_1^s = 0.45ml/\mu g$ $\kappa_1^a = 0.81ml/\mu g$, $\kappa_1^b = 0.41ml/\mu g$ $\kappa_1^{ab} = 0.69ml/\mu g$
$\tilde{\kappa}_1^*$	Maximal growth inhibition of bacterial type $*$ by antibiotic B	$\tilde{\kappa}_1^c = 0.56ml/\mu g$, $\tilde{\kappa}_1^s = 0.47ml/\mu g$ $\tilde{\kappa}_1^a = 0.38ml/\mu g$, $\tilde{\kappa}_1^b = 0.79ml/\mu g$, $\tilde{\kappa}_1^{ab} = 0.71ml/\mu g$
κ_2^*	Affinity for antibiotic A of bacterial type $*$	$\kappa_2^c = 0.15ml/\mu g$, $\kappa_2^s = 0.2ml/\mu g$, $\kappa_2^a = 0.3ml/\mu g$, $\kappa_2^b = 0.2ml/\mu g$, $\kappa_2^{ab} = 0.4ml/\mu g$
$\tilde{\kappa}_2^*$	Affinity for antibiotic B of bacterial type $*$	$\tilde{\kappa}_2^c = 0.18ml/\mu g$, $\tilde{\kappa}_2^s = 0.25ml/\mu g$, $\tilde{\kappa}_2^a = 0.2ml/\mu g$, $\tilde{\kappa}_2^b = 0.4ml/\mu g$, $\tilde{\kappa}_2^{ab} = 0.39ml/\mu g$

Supplement fig. 5 – This shows a theoretical 8-bed ward where 6 different antibiotic policies are simulated on a queue of 100 patients using equation (27) and parameters in Table 3. The leftmost plot of each panel shows the moment a patient is assigned to a bed (black lines) and which drug is prescribed for each patient at every instance in time (blue corresponds to drug A, green to drug B and white when no drug is given). Health states of each patient are shown in the centre plot (red denotes high levels of infection, yellow intermediate pathogen densities and white good health). The relative frequency of the two single-drug resistant pathogens in each patient is shown in the right (grey boxes illustrate patients carrying equal fractions of two different single-drug resistant pathogens, green and blue denote A and B resistant pathogens). (a) Hospital dynamics when no-one is treated. (b) **Empirical treatment**. (c) **Periodic cycling** with an optimal cycling cadence of 7 days. (d) **Periodic Antimicrobial Monitoring and Supervision** (PAMS). (e) **Surveillance-based rotation**. (f) **DNA diagnosis-based treatment**.

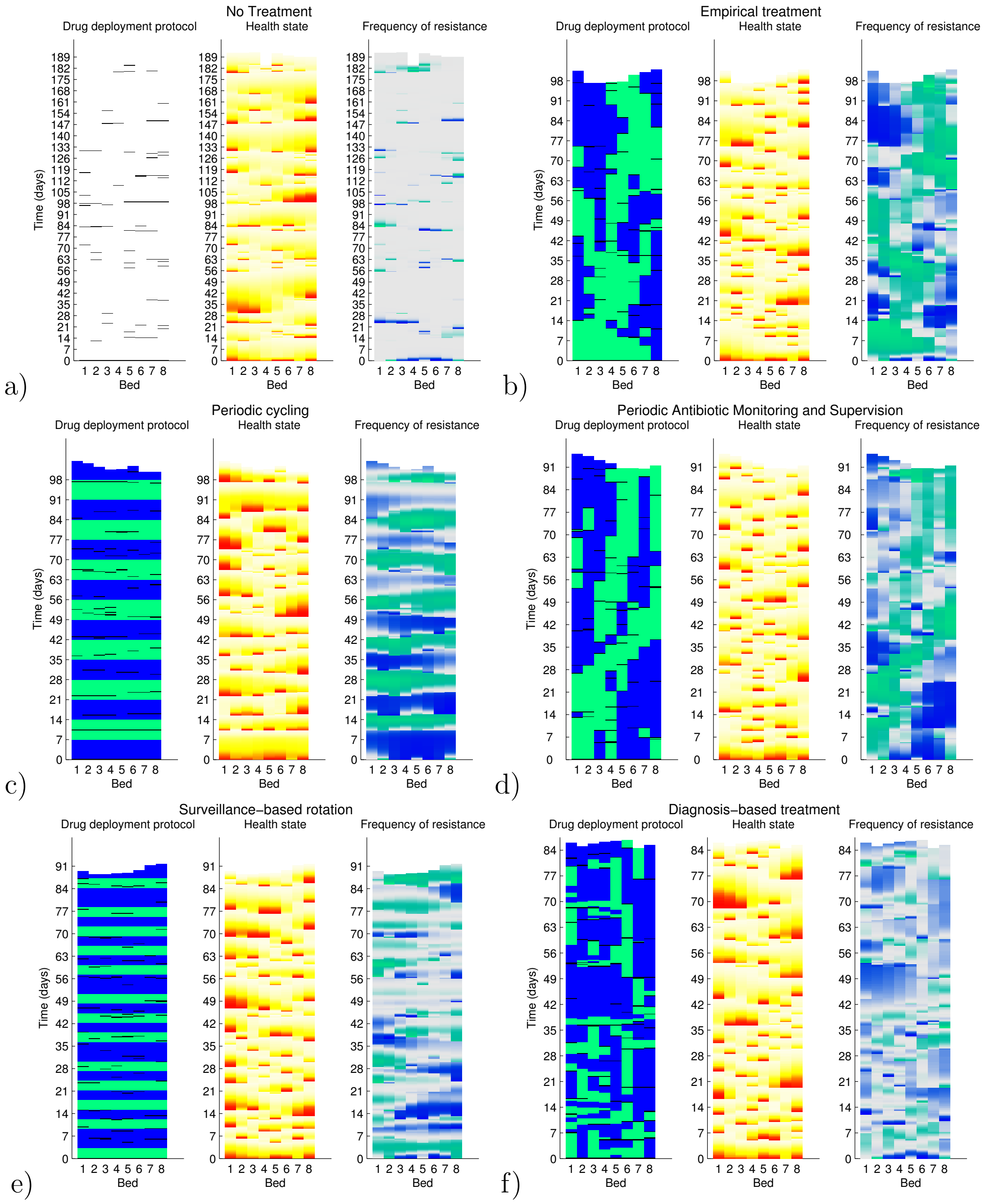

Supplement fig. 6 – The replacement scenario for community-acquired resistance. This figure shows the initial densities of within-host commensal and pathogenic bacteria for a queue of 100 patients (ordered in different ways in each column, as indicated) and the probability that a patient is infected by a single drug-resistant mutant is controlled by a parameter $0 < \rho < 1$ as described in the text. (left column) This shows the initial bacterial densities for each patient in the queue where the colours denote pathogens and black denotes the commensal density. (right column) This shows the initial frequency of single-drug and multi-drug resistant pathogens. (a) This is the case where no drug resistance is observed in the community, so that $\rho = 0$. (c) This is where intermediate levels of resistance are observed in the community, so $\rho = 0.5$. (c) Finally, high levels of resistance are seen in the community, $\rho = 1$.

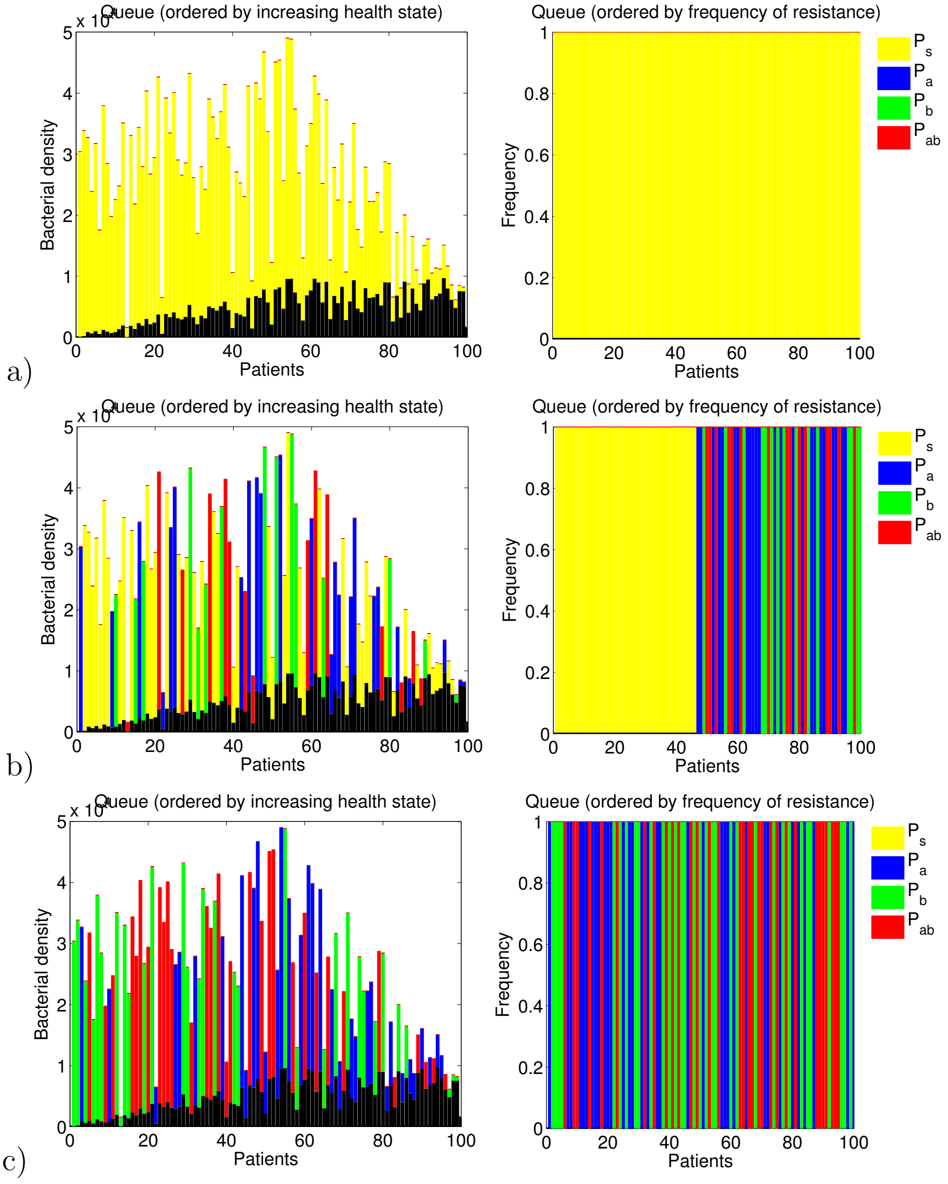

Supplement fig. 7 – The addition scenario for community-acquired resistance. In this case patients are infected by random densities of susceptible, single-drug and multi-drug resistant pathogens coexisting in the host before treatment is initiated and, just as Supplement fig. 6, the parameter $0 < \rho < 1$ controls the frequency of resistance in the community described in the text. (left column) This shows the initial bacterial densities in patients in the queue where the colours denote pathogens and black denotes the commensal density. (right column) This shows frequencies of susceptible, single-drug and multidrug resistant pathogens in each patient. (a) This is the case where no drug resistance is observed in the community, so $\rho = 0$. (b) This is where intermediate levels of resistance are seen in the community, so $\rho = 0.5$. (c) Finally, this shows high levels of resistance in the community, meaning $\rho = 1$.

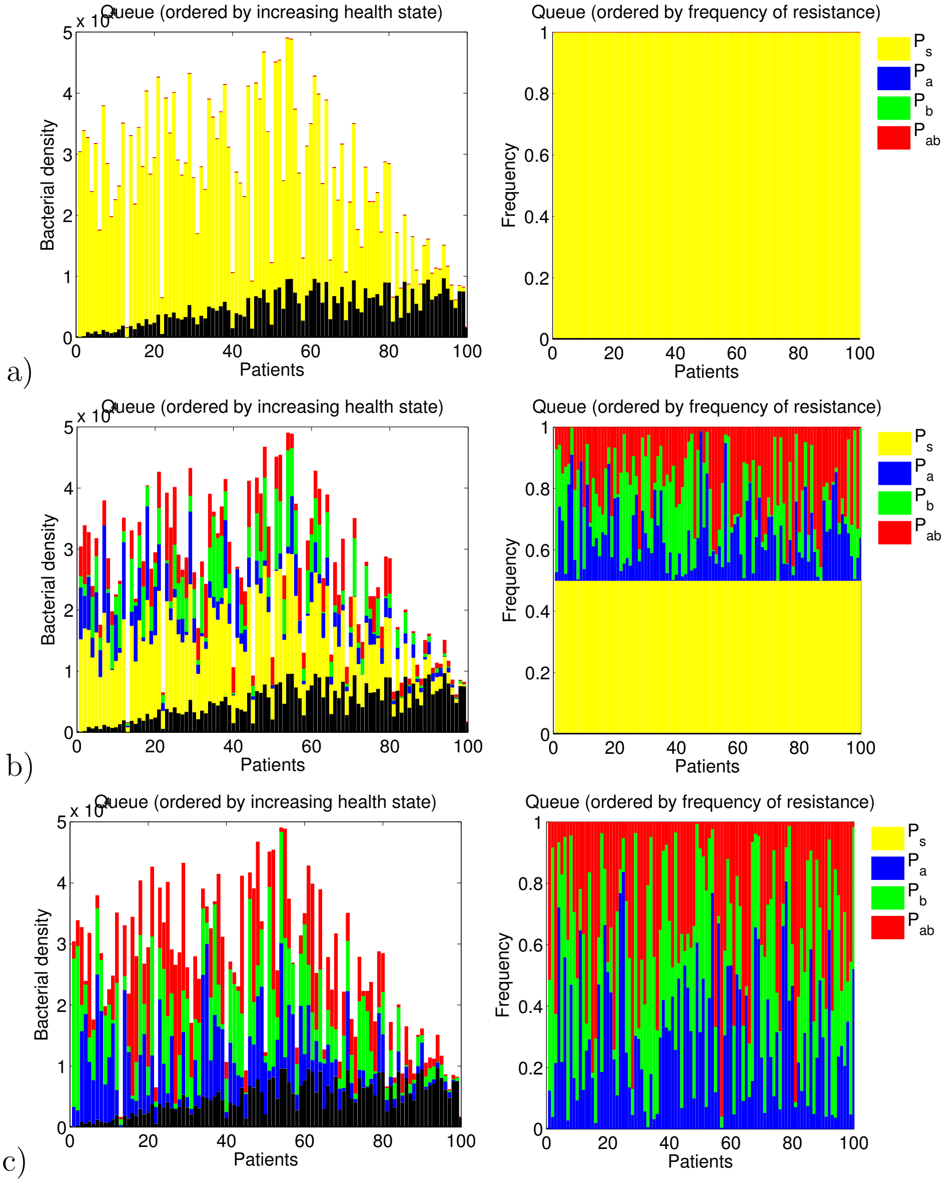

References

[1] R. Beardmore and R. Pena-Miller. Antibiotic cycling versus mixing: the difficulty of using mathematical models to definitively quantify their relative merits. *Math Biosci Eng*, 7(4):923–933, Oct 2010.

[2] R. Beardmore and R. Pena-Miller. Rotating antibiotics selects optimally against antibiotic resistance, in theory. *Mathematical Biosciences and Engineering*, 7(3):527–552, 2010.

[3] C. T. Bergstrom, M. Lo, and M. Lipsitch. Ecological theory suggests that antimicrobial cycling will not reduce antimicrobial resistance in hospitals. *PNAS*, 101:13285–13290, 2004.

[4] Jessica M. A. Blair, Vassiliy N. Bavro, Vito Ricci, Niraj Modi, Pierpaolo Cacciotto, Ulrich Kleinekathoefer, Paolo Ruggerone, Attilio V. Vargiu, Alison J. Baylay, Helen E. Smith, Yvonne Brandon, David Galloway, and Laura J. V. Piddock. Acrb drug-binding pocket substitution confers clinically relevant resistance and altered substrate specificity. *Proceedings of the National Academy of Sciences*, 112(11):3511–3516, 2015.

[5] S Bonhoeffer, M Lipsitch, and B R Levin. Evaluating treatment protocols to prevent antibiotic resistance. *Proc Natl Acad Sci U S A*, 94(22):12106–12111, Oct 1997.

[6] E. M. Brown and D. Nathwani. Antibiotic cycling or rotation: a systematic review of the evidence of efficacy. *J. of Antimicrobial Chemotherapy*, 55:6–9, 2005.

[7] K. C. Chow, X. Wang, and C. Castillo-Chavez. A mathematical model of nosocomial infection and antibiotic resistance: evaluating the efficacy of antimicrobial cycling programs and patient isolation on dual resistance. download from `http://mtbi.asu.edu/downloads/`, 2009.

[8] Desmond J. Higham. An algorithmic introduction to numerical simulation of stochastic differential equations. *SIAM Review*, 43(3):pp. 525–546, 2001.

[9] M.H. Kollef, J. Vlasnik, L. Sharpless, C. Pasque, D. Murphy, and V. Fraser. Scheduled change of antibiotic classes: a strategy to decrease the incidence of ventilator-associated pneumonia. *Am J Respir Crit Care Med*, 1997.

[10] B. Levin and M. Bonten. Cycling antibiotics may not be good for your health. *PNAS*, 101(36):13101–13102, 2004.

[11] Conan MacDougall and Ron E Polk. Antimicrobial stewardship programs in health care systems. *Clinical Microbiology Reviews*, 18(4):638–656, 2005.

[12] Ian M. Mitchell. The flexible, extensible and efficient toolbox of level set methods. *Journal of Scientific Computing*, 35(2–3):300–329, June 2008.

[13] Amy Pakyz, J Patrick Powell, Spencer E Harpe, Chris Johnson, Michael Edmond, and Ron E Polk. Diversity of antimicrobial use and resistance in 42 hospitals in the United States. *Pharmacotherapy*, 28(7):906–912, Jul 2008.

[14] T. C. Reluga. Simple models of antibiotic cycling. *Mathematical Medicine and Biology*, 22(2):187–208, June 2005.
